# The Molecular Quality and Mitochondrial Activity of Porcine Cumulus–Oocyte Complexes Are Affected by Their Exposure to Three Endocrine-Active Compounds under 3D In Vitro Maturation Conditions

**DOI:** 10.3390/ijms23094572

**Published:** 2022-04-20

**Authors:** Gabriela Gorczyca, Kamil Wartalski, Marek Romek, Marcin Samiec, Małgorzata Duda

**Affiliations:** 1 Department of Endocrinology, Institute of Zoology and Biomedical Research, Faculty of Biology, Jagiellonian University in Krakow, Gronostajowa 9 Street, 30-387 Krakow, Poland; gabriela.gorczyca@doctoral.uj.edu.pl; 2 Department of Histology, Jagiellonian University Medical College, Kopernika 7 Street, 31-034 Krakow, Poland; kamil.wartalski@uj.edu.pl; 3 Department of Cell Biology and Imaging, Institute of Zoology and Biomedical Research, Faculty of Biology, Jagiellonian University in Krakow, Gronostajowa 9 Street, 30-387 Krakow, Poland; marek.romek@uj.edu.pl; 4 Department of Reproductive Biotechnology and Cryoconservation, National Research Institute of Animal Production, Krakowska 1 Street, 32-083 Balice, Poland

**Keywords:** pig, oocyte, cumulus cells, 3D in vitro maturation, endocrine-active compounds, molecular quality, apoptotic cell death, mitochondria, mitophagy

## Abstract

Thus far, the potential short- and long-term detrimental effects of a variety of environmental chemicals designated as endocrine-active compounds (EACs) have been found to interfere with histo- and anatomo-physiological functions of the reproductive system in humans and wildlife species. For those reasons, this study sought to examine whether selected EACs, which encompass the fungicide vinclozolin (Vnz), the androgenic anabolic steroid nandrolone (Ndn) and the immunosuppressant cyclosporin A (CsA), affect the developmental competence and molecular quality (MQ) of porcine cumulus–oocyte complexes (COCs) subjected to in vitro maturation (IVM) under 3D culture conditions. The COCs underwent 3D-IVM in the presence of Vnz, Ndn or CsA for 48 h. To explore whether the selected EACs induce internucleosomal DNA fragmentation in cumulus cells (CCs), TUNEL-assisted detection of late apoptotic cells was performed. Additionally, for the detailed evaluation of pro- and antiapoptotic pathways in COCs, apoptosis proteome profiler arrays were used. To determine changes in intracellular metabolism in COCs, comprehensive assessments of mitochondrial ultrastructure and activity were carried out. Moreover, the relative abundances (RAs) of mRNAs transcribed from genes that are involved in scavenging reactive oxygen species (ROS), such as *SIRT3* and *FOXO3*, and intramitochondrial bioenergetic balance, such as ATP synthase subunit (*ATP5A1*), were ascertained. Finally, to investigate the extent of progression of oocyte maturation, the intraooplasmic levels of cAMP and the RAs of mRNA transcripts encoding regulatory and biocatalytic subunits of a heterodimeric meiosis-promoting factor, termed cyclin B1 (*CCNB1*) and cyclin-dependent kinase 1 (*CDC2*), were also estimated. The obtained results provide, for the first time, strong evidence that both Vnz and Ndn decrease the developmental competence of oocytes and stimulate apoptosis processes in CCs. The present study is also the first to highlight that Vnz accelerates the maturation process in immature oocytes due to both increased ROS production and the augmented RA of the *CCNB1* gene. Furthermore, Vnz was proven to trigger proapoptotic events in CCs by prompting the activity of the FOXO3 transcription factor, which regulates the mitochondrial apoptosis pathway. In turn, Ndn was shown to inhibit oocyte maturation by inducing molecular events that ultimately lead to an increase in the intraooplasmic cAMP concentration. However, due to the simultaneous enhancement of the expression of TNF-β and HSP27 proteins in CCs, Ndn might be responsible for the onset of their neoplastic transformation. Finally, our current investigation is the first to clearly demonstrate that although CsA did not interfere with the nuclear and cytoplasmic maturation of oocytes, by inducing mitophagy in CCs, it disrupted oocyte metabolism, consequently attenuating the parameters related to the MQ of COCs. Summing up, Vnz, Ndn and CsA reduced not only the processes of growth and IVM but also the MQ of porcine COCs, which might make them unsuitable for assisted reproductive technologies (ARTs) such as in vitro fertilization by either gamete co-incubation or intracytoplasmic sperm injection (ICSI) and cloning by somatic cell nuclear transfer (SCNT).

## 1. Introduction

The presence of various industrial contaminants, pesticides and fungicides in the environment and the frequent abuse of anabolic steroids or hormonal replacement therapy drugs have led to the scientific interest in a group of chemicals called endocrine-active compounds (EACs) or endocrine-disrupting chemicals (EDCs) [1]. EACs/EDCs disturb the normal function of endocrine glands and the hormonal balance of organisms by imposing biological effects, e.g., by binding with receptors for endogenous hormones. Not without significance is the high bioaccumulation of these compounds in plant and animal tissues, which indirectly, through their consumption, may influence organisms and people of both sexes [2]. Among potent EACs/EDCs, mention should be made of: (1) a commonly used dicarboximide fungicide designated as vinclozolin (Vnz); (2) a member of the androgenic anabolic steroid (AAS) family, known as nandrolone (Ndn); and (3) a representative of immunosuppressants termed cyclosporin A (CsA).

Vnz is a component of anti-gray mold preparations used in the protection of crops of fruits and vegetables. Two major ring-opened metabolites of Vnz were detected in rodent physiological fluids and tissue extracts following in vivo exposure [3]. Since Vnz possesses anti-androgenic activity in both fish and mammals [4,5,6], exposure to it during the gonadal sex determination period promotes a transgenerational increase in pregnancy abnormalities and female adult-onset malformations in reproductive organs [7,8]. Our previous studies have shown that vinclozolin at an environmentally relevant concentration might contribute to the amplification and propagation of apoptotic cell death in the granulosa layer, leading to the rapid removal of atretic follicles in the porcine ovary. Additionally, it seems possible that vinclozolin activates nongenomic signaling pathways, directly modifying the androgen receptor action [9,10,11,12].

Another interesting group of EACs is AASs, which encompass biochemical substances synthesized from testosterone or one of their derivatives. Depending on the target tissue, AASs exhibit anabolic or androgenic effects [13,14]. AASs are clinically indicated, for example, for the treatment of chronic obstructive pulmonary disease, for the treatment of hepatic or renal failure and in conditions of either acquired immunodeficiency syndrome (AIDS) or carcinogenesis, tumor progression and metastasis [15,16,17]. AASs are also recommended for androgen replacement therapy after menopause [18,19]. A cause for concern is the increasing illegal use of AASs by athletes and amateurs [20,21,22,23]. This is unfortunately directly caused by the dissemination of the image of strong bodies as the model for the ideal posture in mass communication media [24]. Usually, AASs are administered at supraphysiological doses [25], which are 5-fold to 29-fold higher than the dose recommended for hormonal replacement therapy. Because of the abusive use of AASs by women, numerous side effects, such as atrophy of the breasts, aggressiveness and menstrual irregularity, were found [26,27]. The observed alterations were dose- and time-dependent [28]. Moreover, in women abusing AASs, abnormal gonadal function, such as precocious/accelerated or delayed puberty, anovulation or luteal phase deficiency, may occur [25]. Unfortunately, some of these changes are irreversible even after cessation of AAS administration [29]. As a further matter, AASs, despite regulations (the European Community banned the use of anabolic substances in Europe by means of laws 96/22/EC and 96/23/EC), are still used for anabolic purposes in industrial livestock breeding [30]. Among AAS derivatives, Ndn is the most used injectable steroid [31]. Ndn, acting through androgen receptors, has a strong anabolic and weak androgenic effect [32]. Additionally, Ndn is a strong progestogen [32]. In medicine, Ndn is used in convalescence after debilitating diseases and to improve the physical condition of the body [14,33]. It is also known that the pharmacological dose of Ndn slows cell growth, inhibits mitochondrial respiration, inhibits respiratory chain complexes I and III, and increases the production of mitochondrial reactive oxygen species (ROS). Chronic administration of Ndn favors the maintenance of stem cells in various tissues, but it may simultaneously increase the risk of their neoplastic transformation [34,35]. Several recent research results also indicate that Ndn negatively affects the functioning of the female reproductive system [36,37,38,39].

CsA is a cyclic peptide produced by fungi belonging to the species *Tolypocladium inflatum* [40]. Due to its natural immunosuppressive properties, CsA is used in transplant medicine. Most often, it is administered orally; it is absorbed in the intestines and is metabolized in the liver by cytochrome P450 enzymes [41]. The mechanism of action of CsA is based on the blocking of the early stages of T cell activation. This results in a delay in antibody production and macrophage activation [42], which reduces the risk of graft rejection [43]. Due to its ability to interact with membrane proteins, CsA is one of the most used compounds in studies targeting the exploration of mitochondrial function [44]. In canine cardiomyocytes, CsA has been shown to protect mitochondria by blocking the opening of mitochondrial permeability transition pores (mPTPs). Opening even a single mPTP can cause depolarization of the mitochondrial membrane and vacuolization of the mitochondria; it is one of the signals that activate the processes of programmed cell death [45]. Additionally, CsA has been found to increase the membrane potential of mitochondria [46]. Studies aiming to analyze CsA-mediated effects on ovulation processes in rats have demonstrated that CsA triggers a decline in the quantity of mature antral ovarian follicles displaying the ability to ovulate and, consequently, a reduction in the assumed number of ovulated oocytes [47]. In turn, exposure of pregnant mice to CsA at doses of 20 and 30 mg/kg reduced the possibility of embryo implantation [48]. Although the harmful impacts of EACs on female reproductive parameters have already been extensively recognized, only a few studies have focused on the elucidation of the mechanisms underlying the actions of the above-mentioned EACs that are exerted on oocytes and the surrounding cumulus cells (CCs) [49,50].

Oocyte maturation is the culmination of an extended period of their growth and development within the ovarian follicle. Fully grown oocytes become competent to undergo three synergistic processes of maturation: nuclear, epigenomic and cytoplasmic, which should be initiated and completed synchronously. Whereas nuclear maturation encompasses the release of oocytes from dictyotene (i.e., germinal vesicle/GV stage)-related meiotic arrest and the progression of meiosis from prophase I to metaphase II (MII) [51,52], their epigenomic counterpart depends on the onset, progression and termination of epigenetic covalent modifications in terms of the extent of methylation within genomic DNA cytosine residues and associated alterations in deacetylation and methylation/demethylation profiles within histone lysine and arginine moieties of chromatin nucleosomal cores [53,54,55]. In turn, cytoplasmic maturation involves the accumulation of mRNA, proteins and nutrients and the redistribution of organelles that are cumulatively indispensable to attain meiotic competence, efficiently finalize meiotic maturation and finally render MII-stage oocytes able to either be successfully fertilized under in vivo or in vitro conditions [56,57,58,59,60] or be effectively subjected to the procedures of cloning by somatic cell nuclear transfer (SCNT) [61,62,63].

It is noteworthy that the occurrence of morphologically, ultrastructurally and cytophysiologically normal ovarian follicle-derived compartments composed of CCs that are characterized by high molecular quality seems to be extremely important for the faithful progression and termination of oocyte maturation [64,65,66]. Intrafollicular compartments composed of CCs and oocytes have been found to reciprocally cooperate in order to bi-directionally exchange small molecules (e.g., adenosine triphosphate (ATP), cyclic adenosine 3′,5′-monophosphate (cAMP) and calcium ions), which takes place through gap junctions [67,68]. The proper molecular function of CCs allows for maintaining oocyte meiotic arrest, followed by participation in meiosis resumption, and supports ooplasmic maturation. This bi-directional interplay between oocyte- and CC-specific intraovarian compartments is crucial for perpetuating not only synergistic interrelations but also proper coordination of the molecular network necessary for transcriptional and proteomic crosstalk between intrafollicular female gamete- and cumulus oophorus-committed niches [69].

Another pivotal factor on which the adequate growth and maturation of oocytes directly depend is their mitochondrial bioenergetic reserve capacity [70,71]. On the one hand, the mitochondria represent the powerhouse of each cell, and therefore, they are designated as so-called bioaccumulators or intracellular deposits of bioenergy arising from ATP synthesis [72,73]. On the other hand, these organelles are also responsible for not only regulating oxidation–reduction (redox) reactions but also retaining Ca^2+^ homeostasis and controlling apoptosis processes [74,75].

Taking into account the modern strategies of assisted reproductive technologies (ARTs) and in vitro embryo production (IVP), the absence of mitochondrion-deficient cells and the intracellular imbalance of mitochondrial compartments, followed by the lack of chronic cytophysiological failures/insufficiencies of mitochondria, have been shown to play a key role in the determination of high parameters of efficiency for the processes of in vitro maturation (IVM), in vitro fertilization (IVF), SCNT-mediated reconstruction of enucleated oocytes and preimplantation development of embryos generated by IVF or SCNT-based cloning [76,77,78,79,80,81]. For those reasons, the high incidence of perturbations in the quantity and function of mitochondria in oocytes may reduce their quality and subsequently compromise embryonic development [82,83,84]. Therefore, it is so important to thoroughly accomplish the molecular characterization of intracellular niches committed to/occupied by mitochondria and comprehensively unravel the transcriptional and proteomic alterations leading to intramitochondrial dysfunctions and disturbances in intermitochondrial communication following the exposure of oocytes to the selected EACs. If such exposure to ectopic or environmental endocrine disruptors induces ultrastructural damage and cytophysiological incompetence of mitochondria, it may contribute to the rapid diminishment of oocyte quality and consequently to the depletion of the intraovarian reserve of female gametes and the progressive scarcity of meiotically competent oocytes retaining enhanced fertilizability [85,86,87,88,89] or capability to be reconstructed by SCNT [90,91,92].

In light of the above, our research hypothesis assumed that in porcine cumulus oocyte–complexes (COCs), Vnz, Ndn and CsA adversely affect the processes of growth and IVM, which might make them unsuitable for ARTs such as standard IVF by gamete co-incubation or microsurgical IVF by intracytoplasmic sperm injection (ICSI) and SCNT-based cloning. Thus, the aim of this study was to investigate the impacts of the selected EACs on parameters associated with the molecular quality of COCs subjected to the IVM procedure with the use of a three-dimensional (3D) culture model. A multi-faceted assessment of the molecular quality of 3D-IVM-generated COCs was carried out by estimating their transcriptional and proteomic profiles based on the induction of intracellular pro- and antiapoptotic pathways. To the best of our knowledge, the current investigation is the first to holistically demonstrate and exhaustively track the Vnz-, Ndn- and CsA-dependent mechanisms by which these endocrine disruptors can evoke rapid and robust alterations in transcriptional and proteomic signatures in porcine 3D-IVM-derived oocytes. The present research also provides, for the first time, a collective demonstration that genome- and proteome-wide scales of these alterations induce the strong multipath propagation of apoptotic cell death-related signals, irreversibly leading to a drastic attenuation of the molecular quality of pig oocytes subjected to extracorporeal meiotic maturation in the 3D model. Additionally, changes in the molecular quality of COCs undergoing IVM under 3D culture conditions were confirmed by identifying not only alterations in the cytosolic levels of cAMP but also the occurrence of ultrastructural transformations and late-apoptotic symptoms of internucleosomal DNA fragmentation in TUNEL-positive oocytes and surrounding CCs. Moreover, the current investigation sought to evaluate in detail the Vnz-, Ndn- and CsA-mediated effects exerted on COCs’ mitochondrial compartments, which were reflected in increases in: (1) defects in mitochondrion-specific morphology; (2) dysfunctions detected in mitochondrial activity and intraorganelle cytometabolic balance; (3) impairments in the distribution of mitochondria and interorganelle signal transduction (i.e., organelle interactions) between mitochondrial compartments; (4) disturbances in ATP production; and (5) dysregulations in the expression of mitophagy-related markers.

## 2. Results

### 2.1. Vinclozolin and Nandrolone Accelerate Apoptosis of Cumulus Cells

**Figure 1:**
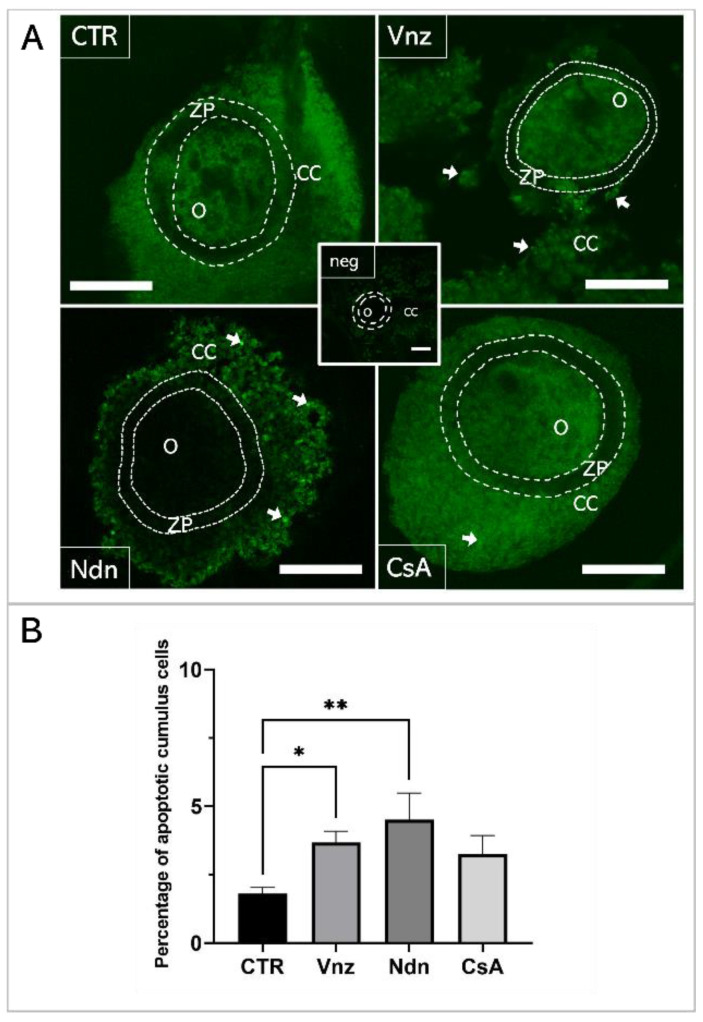
(**A**): TUNEL assay-mediated identification of the localization of late-apoptotic cells in porcine COCs subjected to the IVM procedure using a 3D culture model (alginate capsules). Representative images of COCs stemming from control (CTR) and experimental groups of 3D-IVM: in the presence of vinclozolin (Vnz), nandrolone (Ndn) or cyclosporin A (CsA). The contours of the oocytes are marked with dashed lines. White arrows indicate TUNEL-positive late-apoptotic cumulus cells (CCs) that fluoresced in bright green. A negative control was performed without active terminal deoxynucleotidyl transferase (TdT) enzyme (neg). O: oocyte; ZP: zona pellucida. All scale bars represent 100 μm (magnification 40×). (**B**): The results of the semi-quantitative TUNEL analysis. The results represent the mean value with *n* = 9 ± standard deviation (SD) for all experimental groups. Statistical analysis: homogeneity of variance—Brown–Forsythe test; normality of distribution—Bartlett’s test and one-way ANOVA followed by Dunnett’s post hoc test, * *p* < 0.05, ** *p* < 0.01.

A TUNEL analysis was performed to examine genomic DNA fragmentation as an indicator of late apoptosis in porcine COCs exposed to Vnz, Ndn or CsA during IVM under 3D culture conditions (Figure 1). The presence of apoptotic cells (white arrows in panel A of Figure 1) was observed in the CCs stemming from COCs that represent all three experimental groups. However, the number of TUNEL-positive CCs increased significantly after exposure to Ndn (4.5% ± 0.97%) and Vnz (3.67% ± 0.41%). Compared to the control (CTR) group (1.79% ± 0.19%), these differences were statistically significant (*p* < 0.05 and *p* < 0.01, respectively) (Figure 1B).

### 2.2. Analysis of the Apoptosis Mechanism in 3D-IVM-Generated Cumulus–Oocyte Complexes Treated with the Selected Endocrine Disruptors

**Figure 2:**
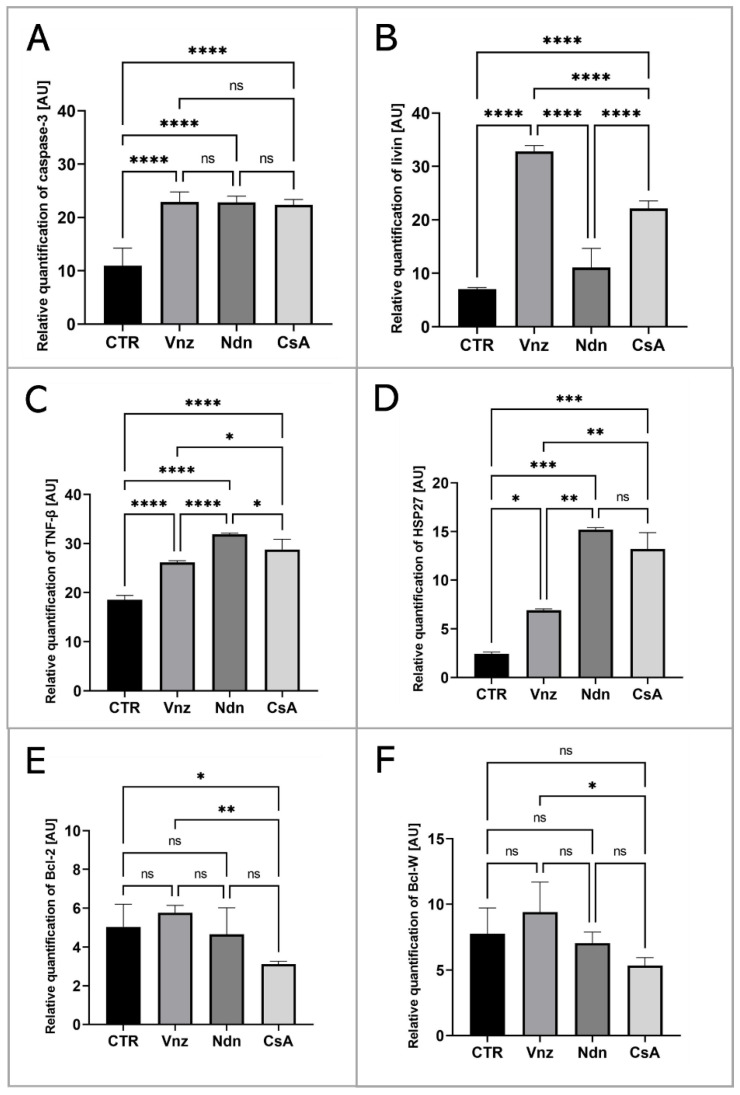
Apoptosis array analysis at the level of total protein in porcine COCs subjected to the IVM procedure using a 3D culture model (alginate capsules) in control cultures (CTR) or in experimental cultures: in the presence of vinclozolin (Vnz), nandrolone (Ndn) or cyclosporin A (CsA). The graphs show the relative content of caspase 3 (**A**), livin (**B**), TNF-β (**C**), HSP27 (**D**), Bcl-2 (**E**) and Bcl-w (**F**) proteins obtained from measurements of the optical density of the spots representing a specific signal after normalization against the positive control. Results represent the mean with *n* = 5 ± standard deviation (SD); each “*n*” consisted of 200 COCs. Statistical analysis: homogeneity of variance—Brown–Forsythe test; normality of distribution—Bartlett’s test and one-way ANOVA followed by Dunnett’s post hoc test, * *p* < 0.05, ** *p* < 0.01, *** *p* < 0.001, **** *p* < 0.0001; ns-non—significant.

**Figure 3:**
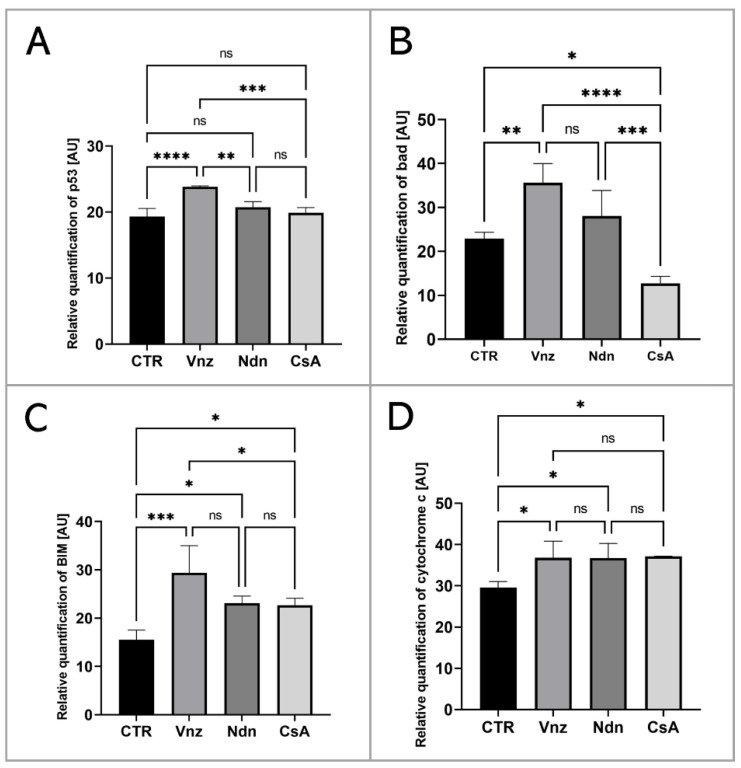
Analysis of the expression of proteins involved in the activation of the mitochondrial pathway of apoptosis at the level of total protein (apoptosis antibody array kit) in porcine COCs subjected to the IVM procedure using a 3D culture model (alginate capsules) in control cultures (CTR) or in experimental cultures: in the presence of vinclozolin (Vnz), nandrolone (Ndn) or cyclosporin A (CsA). The graphs show the relative content of p53 (**A**), bad (**B**), BIM (**C**) and cytochrome c (**D**) proteins obtained from measurements of the optical density of the spots representing a specific signal after normalization against the positive control. Results represent the mean with *n* = 5 ± standard deviation (SD); each “*n*” consisted of 200 COCs. Statistical analysis: homogeneity of variance—Brown–Forsythe test; normality of distribution—Bartlett’s test and one-way ANOVA followed by Dunnett’s post hoc test, * *p* < 0.05, ** *p* < 0.01, *** *p* < 0.001, **** *p* < 0.0001; ns—nonsignificant.

To thoroughly unravel the molecular nature of the increased incidence of programmed cell death in COCs subjected to Vnz, Ndn or CsA treatment, the Human Apoptosis Antibody Array kit was used to analyze the profiles of expression of apoptosis-related proteins. The obtained data showed that there were remarkable enhancements in the expression profiles of caspase-3 (CTR 10.9 ± 3.30, Vnz 22.9 ± 1.90, Ndn 22.8 ± 1.18, CsA 22.3 ± 1.02), tumor necrosis factor-β (TNF-β) (CTR 18.5 ± 0.91, Vnz 26.1 ± 0.42, Ndn 31.9 ± 0.27, CsA 28.8 ± 2.02), heat shock protein (HSP) 27 (CTR 2.4 ± 0.14, Vnz 6.9 ± 0.13, Ndn 15.2 ± 0.24, CsA 13.2 ± 1.68) and livin (CTR 7.0 ± 0.32, Vnz 32.8 ± 1.09, Ndn 11.6 ± 3.60, CsA 22.1 ± 1.42) (Figure 2A–D) in COCs derived from all of the experimental groups compared to CTR, while the expression levels of Bcl-2 and Bcl-w proteins were either downregulated in COCs derived from Ndn (4.7 ± 1.36 and 7.0 ± 0.84 respectively) and CsA (3.1 ± 0.14 and 5.3 ± 0.61 respectively) experimental groups or showed no significant impact (*p* = 0.69), as was observed for COCs derived from the Vnz group (5.7 ± 0.38 and 9.4 ± 2.30, respectively) (Figure 2E,F). Furthermore, the protein array analysis revealed that both Vnz (23.8 ± 1.68, 29.4 ± 5.62, 36.8 ± 4.01), Ndn (20.7 ± 0.87, 23.1 ± 1.48, 36.7 ± 3.55) and CsA (19.9 ± 0.81, 22.7 ± 1.42, 37.1 ± 0.05) brought about significant increases in p53, BIM and cytochrome c protein levels in COCs during the 3D-IVM procedure (Figure 3A,C,D). A statistically significant increase in the expression of Bad protein after exposure to Vnz (35.6 ± 4.39) or Ndn (28.0 ± 5.80) was also found, whereas the exposure of COCs to CsA (12.7 ± 1.59) gave rise to a decrease in the expression of this protein (Figure 3B). Thus, the observed enhancement of BIM protein expression with the simultaneous inhibition of Bcl-2 expression, which were accompanied by an increase in cytochrome c release from mitochondria, provides strong evidence for the predominant activation of the mitochondrial pathway of apoptotic cell death in the 3D-IVM-produced COCs undergoing treatment with the selected EACs.

### 2.3. Gene Expression of Main Apoptosis and Autophagy Mediators

**Figure 4:**
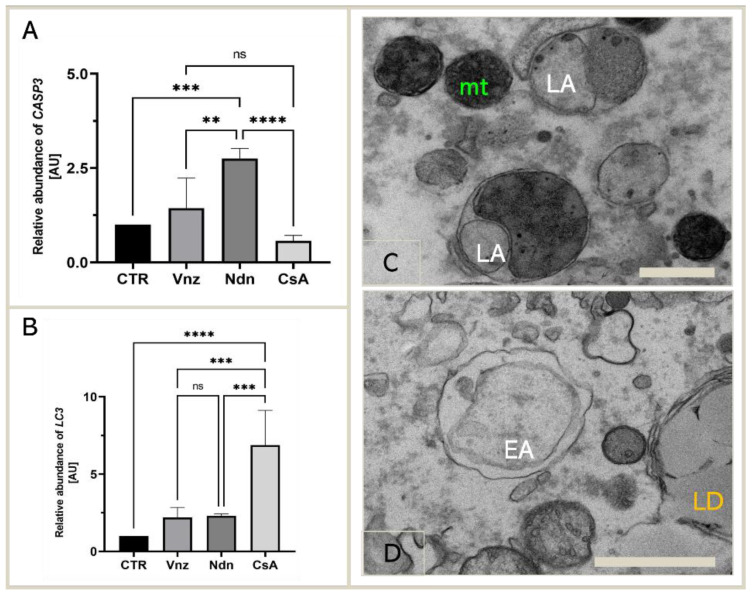
Expression of genes coding for apoptosis and autophagy mediators: CASP3 (**A**) and LC3 (**B**) in porcine COCs subjected to the IVM procedure using a 3D culture model (alginate capsules) in control cultures (CTR) or in experimental cultures: in the presence of vinclozolin (Vnz), nandrolone (Ndn) or cyclosporin A (CsA), shown by RT-qPCR at the transcript level. The results are presented as mean values with *n* = 5 ± standard deviation (SD); each “*n*” consisted of 50 COCs. Statistical analysis: homogeneity of variance—Brown–Forsythe test; normality of distribution—Bartlett’s test and one-way ANOVA followed by Dunnett’s post hoc test, ** *p* < 0.01, *** *p* < 0.001, **** *p* < 0.0001; ns—nonsignificant. (**C**): The ultrastructure of late autophagosome (LA) in oocytes maturing in vitro in the presence of CsA; scale bars represent 400 nm. (**D**): The ultrastructure of early autophagosome (EA) in oocytes maturing in vitro in the presence of Ndn; scale bars represent 900 nm. mt: mitochondria; LD: lipid droplets.

Because the main executioner caspase, caspase-3, was mediated by both Vnz, Ndn and CsA in the protein array analysis, we next examined whether exposure of COCs to selected EACs during 3D-IVM also triggered the modification of caspase-3 expression at the gene level. According to the results acquired using the antibody array, we performed qRT-PCR to detect the expression of *CASP3* mRNA. The obtained results confirmed the significant upregulation of the *CASP3* gene in porcine COCs exposed to Vnz (1.4 ± 0.80) and Ndn (2.8 ± 0.27) during IVM under 3D culture conditions (Figure 4A).

Additionally, considering the finality/irreversibility of apoptosis induction and its consequences for proper oocyte maturation, it was beyond any doubt that these findings must be verified by exploring whether the selected EACs could promote cell survival in the autophagy process via potential interconnections between the apoptotic and autophagy-dependent pathways. For those reasons, qRT-PCR analysis for a pivotal biomarker of autophagy, the *LC3* gene (*MAP1LC3A*), was performed. Quantitatively estimating the relative abundance (RA) of mRNA transcribed from the *MAP1LC3A* gene proved a statistically significant increase (*p* < 0.0001) in the rates of RA of *LC3* transcripts among COCs subjected to 3D-IVM in the presence of CsA (6.9 ± 2.24) as compared to the CTR group. In turn, the quantitative profiles that were identified for *LC3* transcripts in COCs undergoing 3D-IVM and simultaneous treatment with either Vnz (2.2 ± 0.66) or Ndn (2.3 ± 0.12) trended upwards as compared to the control group, but this augmentation did not turn out to be statistically significant (*p* = 0.51 and *p* = 0.42, respectively) (Figure 4B).

The results obtained by quantitatively estimating *LC3* mRNA expression were confirmed by COC ultrastructure analysis (TEM). Numerous late autophagosomes (LAs) were found to occur in the cytoplasm of 3D-IVM-produced oocytes exposed to CsA. LAs contained partially or completely degraded organelle material, which was characterized by a higher electron density than the cytoplasm surrounding the autophagosome. The aforementioned material was most frequently recognizable as mitochondria and peroxisomes (Figure 4C). A lot of early autophagosomes (EAs) were also found to contain ultrastructurally intact cytoplasm that seemed to be identical to the surrounding cytoplasm (Figure 4D). It is noteworthy that the presence of EAs was shown in the cytoplasm of 3D-IVM-generated oocytes derived from Vnz- or Ndn-treated groups.

### 2.4. Cyclosporin A Induces Mitophagy in COCs Undergoing IVM under 3D Culture Conditions

**Figure 5:**
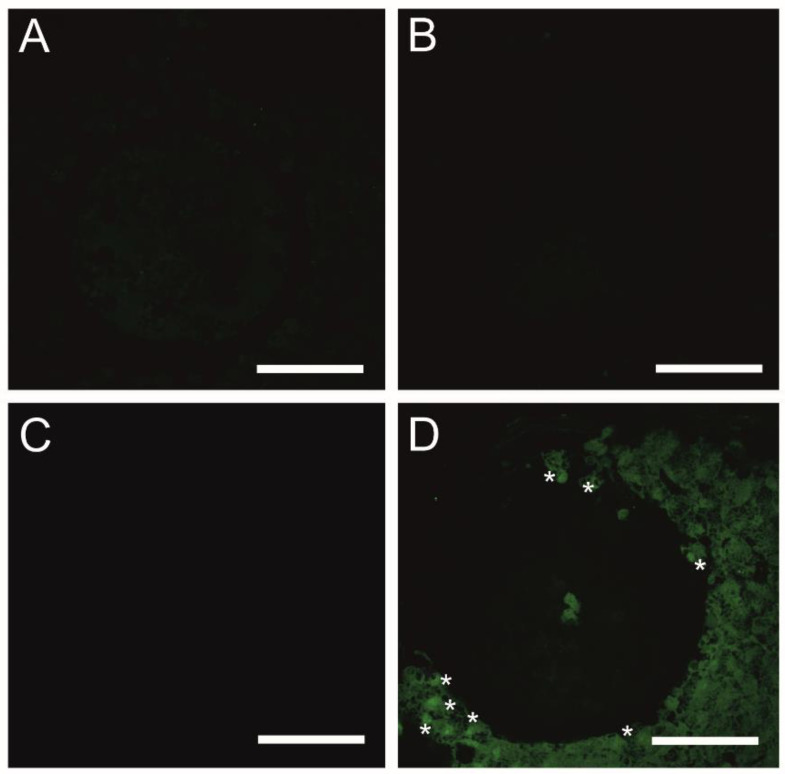
Detection of mitophagy in porcine COCs subjected to the IVM procedure using a 3D culture model (alginate capsules) in control cultures (**A**) or in experimental cultures: in the presence of vinclozolin (**B**), nandrolone (**C**) or cyclosporin A (**D**). White asterisks indicate colocalization of lysosome and mitochondrion. Scale bars represent 50 µm (magnification 40×).

As presented in Figure 5A, the fluorescent image of COCs in the CTR shows that mitophagy was not detected, whereas mitophagy was identified in COCs exposed to CsA during IVM under 3D culture conditions. The identification of mitophagy was reflected in the fusion of lysosomes and the mitochondria (Figure 5D). In COCs treated with Vnz and Ndn, there was no colocalization of mitochondria and lysosomes, which remarkably confirms the lack of mitophagy induction (Figure 5B,C).

### 2.5. Analysis of Cellular Metabolism in 3D-IVM-Generated COCs Treated with the Selected Endocrine Disruptors

To assess the effects of the selected EACs on cellular metabolism in 3D-IVM-derived COCs, the analysis of mitochondria distribution and activity in both CCs and oocytes was performed. Comprehensive efforts were also undertaken to evaluate mitochondrial ultrastructure and explore vital mitochondrial activity in oocytes undergoing exposure to the selected EACs during 3D-IVM. Moreover, quantitative estimation of the transcriptional activities of a panel of genes that encode the ATP synthase subunit (*ATP5A1*) and are involved in the process of scavenging free radicals (*SIRT3* and *FOXO3*) was performed. Additionally, measurements aimed at ascertaining the intracytoplasmic levels of glutathione (GSH) were made.

#### 2.5.1. Analysis of the Distribution and Ultrastructure of Mitochondria in COCs Subjected to 3D-IVM in the Presence of Selected EACs

**Figure 6:**
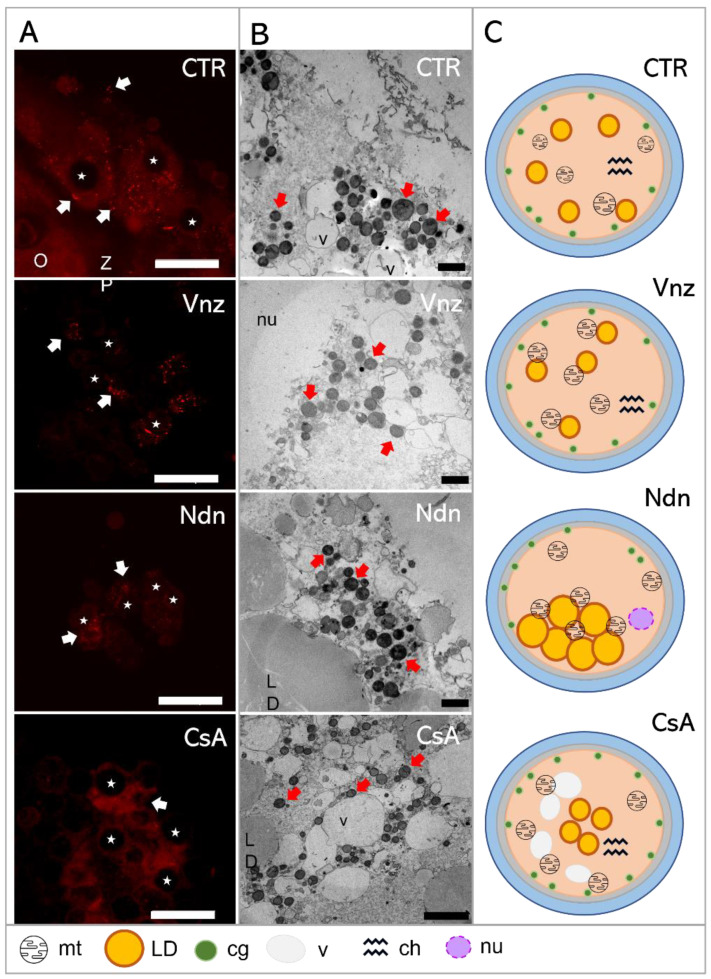
(**A**): Representative images of MitoTracker Orange CM-H2TMRos-positive mitochondrial distribution in porcine CCs subjected to the IVM procedure using a 3D culture model (alginate capsules) in control cultures (CTR) or in experimental cultures: in the presence of vinclozolin (Vnz), nandrolone (Ndn) or cyclosporin A (CsA). (**B**): Transmission electron micrographs of ultrastructure in porcine oocytes subjected to the IVM procedure using a 3D culture model in CTR or in the presence of Vnz, Ndn or CsA. (**C**): A diagram summarizing the results of the ultrastructure analysis of the oocyte and the distribution of organelles in their ooplasm after the completion of 3D-IVM in the presence of selected EACs. O—oocyte; ZP—zona pellucida; LD—lipid droplets; v—vacuole; cg—cortical granules; nu—nucleus; red arrows—mitochondria; stars—nuclei of CCs. Scale bars represent 1000 nm. MtOR magnification 60×.

MitoTracker™ Orange CM-H2TMRos (MtOR) fluorescence staining was used to both visualize the distribution of mitochondria (mt) in CCs and analyze the mitochondrial membrane potential. The results obtained from the fluorescence signal analysis for MtOR detection were compared with the results of the mt ultrastructure analysis using the transmission electron microscope (TEM) (Figure 6).

Based on the observed strong intensity of the fluorescence reaction, high activities of mitochondria in both the cumulus cells and oocyte-specific compartments were confirmed among the 3D-IVM-produced COCs derived from the CTR group (Figure 6A). Generally, the mitochondria of the CCs representing the CTR group were evenly distributed (homogenously dispersed) throughout the cytoplasm. However, in CCs directly adjacent to the oocyte, polar localization of these organelles was found. A vast majority of mt were focused in the pole adjacent to the oocyte, in which mt were concentrated close to the zona pellucida and in the immediate proximity of numerous lipid droplets (Figure 6B). This provides strong evidence for the occurrence of both a cytophysiological network of reciprocal interrelations and functional cooperation between CCs and oocytes and the exchange of substances indispensable to achieve the developmental competence of oocytes (Figure 6C).

In COCs undergoing 3D-IVM in the presence of Vnz, the most intense fluorescence signals for MtOR were found as compared to the other experimental groups and CTR (Figure 6A). In the vast majority of CCs, mitochondria were characterized by perinuclear location; however, in a few cases, mitochondria were focused similarly to those from the control group—at the pole facing the oocyte. Ultrastructural analysis of oocytes from the Vnz group revealed the central location of mostly round mitochondria with a bright matrix (Figure 6B,C).

Among 3D-IVM-derived COCs exposed to Ndn, a significantly weaker fluorescence signal for MtOR was found, which proves the lower activity of mt (Figure 6A). In cumulus cells, mitochondria were located unevenly in the form of single clusters within the cytoplasm. The oocyte mitochondria were small and round with a dark matrix. They were also unevenly distributed between the large lipid droplets (Figure 6B,C).

Porcine COCs subjected to the 3D-IVM procedure in the presence of CsA displayed an even, strong fluorescence signal for MtOR in the cytoplasm of cumulus cells, which proves their high activity (Figure 6A). The distribution of mt in CCs adjacent to the oocyte was polar: i.e., mt were concentrated on the pole facing the oocyte. Ultrastructural analysis of oocytes originating from the CsA group revealed an even, subcortical distribution of mt, which colocalized with numerous vacuoles. The mitochondria were round with a single mitochondrial crest (Figure 6B,C).

#### 2.5.2. The Live Cell-Based Analysis of Mitochondrial Activity in 3D-IVM-Derived COCs Treated with the Selected Endocrine Disruptors

**Figure 7:**
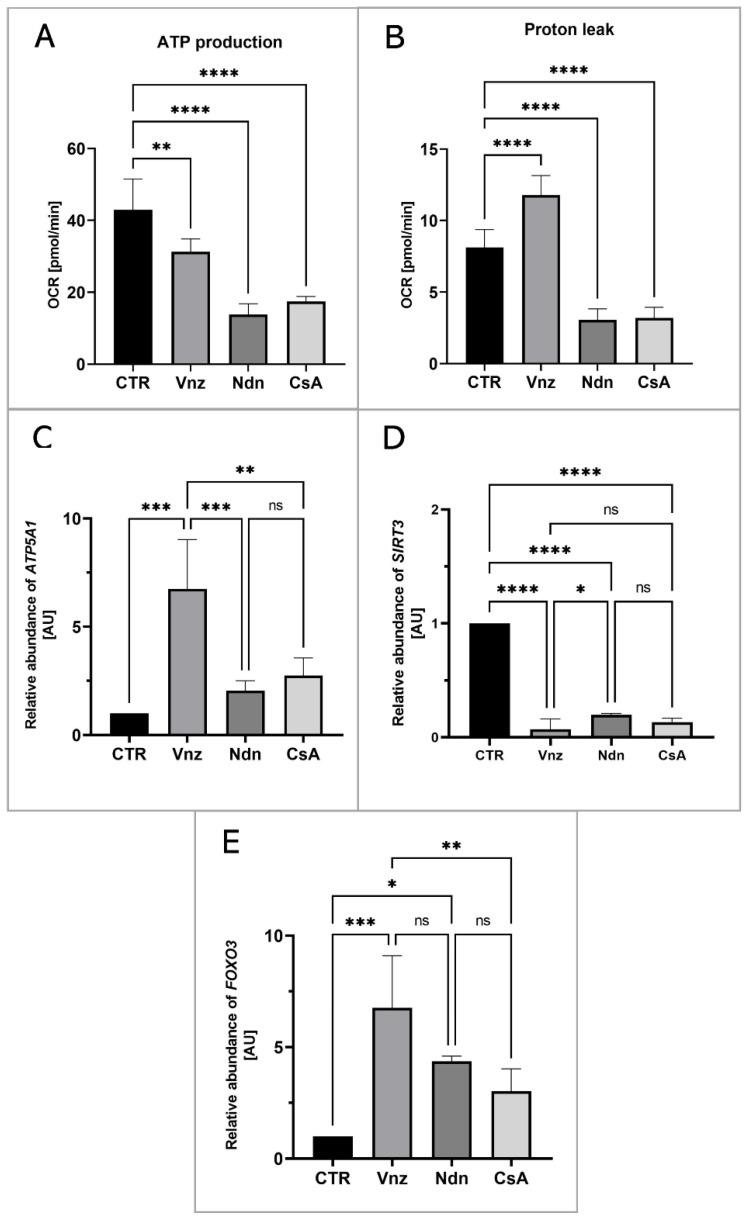
The live cell-based analysis of mitochondrial activity in porcine COCs subjected to the IVM procedure using a 3D culture model (alginate capsules) in control cultures (CTR) or in experimental cultures: in the presence of vinclozolin (Vnz), nandrolone (Ndn) or cyclosporin A (CsA). (**A**): ATP synthesis level analysis performed with the aid of Seahorse XF Cell Mito Stress Test; (**B**): proton leak analysis performed with the aid of Seahorse XF Cell Mito Stress Test; the results represent the mean value with *n* = 5 ± standard deviation (SD), and each “*n*” consisted of 12 COCs. Statistical analysis: homogeneity of variance—Brown–Forsythe test; normality of distribution—Bartlett’s test and one-way ANOVA followed by Dunnett’s post hoc test, ** *p* < 0.01, **** *p* < 0.0001. (**C**–**E**): Quantitative profiles for expression of *ATP5A1*, *SIRT3* and *FOXO3* genes, shown by RT-qPCR at the transcript level. The results represent the mean value with *n* = 5 ± standard deviation (SD), and each “*n*” consisted of 50 COCs. Statistical analysis: homogeneity of variance—Brown–Forsythe test; normality of distribution—Bartlett’s test and one-way ANOVA followed by Dunnett’s post hoc test, * *p* < 0.05, ** *p* < 0.01, *** *p* < 0.001, **** *p* < 0.0001; ns—nonsignificant.

To measure the mitochondrial activity in live cells, the COCs were subjected to the IVM procedure with Seahorse XFp PDL Cell Culture Miniplates under the influence of selected EACs. The Seahorse XF Cell Mito Stress Test was used to analyze ATP production and proton leak in COCs from both CTR and experimental groups (Vnz, Ndn and CsA). In all experimental groups, there was a decline in the level of ATP production (Figure 7A) as compared to CTR (43.0 ± 8.52). The highest decrease was observed in the subpopulations of 3D-IVM-generated COCs exposed to Ndn (3.8 ± 2.98; *p* < 0.0001). At the same level of statistical significance (17.4 ± 1.41; *p* < 0.0001), a diminishment in ATP production was observed for COCs subjected to 3D-IVM in the presence of CsA. In turn, porcine COCs that underwent 3D-IVM and simultaneous Vnz (31.3 ± 3.58) treatment exhibited a reduction in ATP biosynthesis as compared to CTR at a significance level of *p* < 0.01.

A high increase in proton leak (Figure 7B) was found in COCs undergoing 3D-IVM in the presence of Vnz (11.8 ± 1.36). Compared to the control group, this difference was statistically significant (*p* < 0.001). The extent of proton leak in porcine COCs subjected to 3D-IVM and simultaneously exposed to either Ndn (3.1 ± 0.78) or CsA (3.2 ± 0.73) was proven to be 2-fold lower than that estimated for the CTR group (8.1 ± 1.25). This decline turned out to be statistically significant (*p* < 0.0001).

To confirm whether and to what extent the selected EACs bias the efficiency of the ATP synthesis process, the quantitative profile of mRNA transcribed from the *ATP5A1* gene encoding for the ATP catalytic synthase subunit was examined. The results are depicted in Figure 7C. Quantitatively analyzing the RA identified for *ATP5A1* mRNA revealed its 7-fold increase in 3D-IVM-produced COCs treated with Vnz (6.7 ± 2.28) as compared to CTR. This intergroup variability was statistically significant (*p* < 0.0001). In contrast, there were no statistically significant differences (*p* = 0.63 and *p* = 0.24) in the levels of *ATP5A1* mRNA expression in 3D-IVM-derived COCs cultured under the conditions of either Ndn (2.1 ± 0.46) or CsA (2.7 ± 0.81) treatments as compared to the EAC-untreated group.

Additionally, in order to assess whether the selected EACs used in the presented studies activate the cell’s defense mechanisms against ROS, the rates of RA observed for mRNA transcripts that are synthesized from genes involved in scavenging ROS were estimated. The following genes were selected for the analyses: (1) *SIRT3* encoding the sirtuin 3 protein, which is localized in mitochondria and is responsible for the elimination of ROS, and (2) *FOXO3* encoding the FOXO transcription factor, which, together with sirtuin 3, contributes to enhancing the antioxidative activity of mitochondria. As is depicted in Figure 7D, the analysis of the mRNA expression level for the *SIRT3* gene revealed its nearly 10-fold decrease in all experimental groups (Vnz 0.1 ± 0.09, Ndn 0.2 ± 0.01, CsA 0.1 ± 0.03) as compared to the CTR counterpart. This difference was shown to be statistically significant (*p* < 0.0001). In contrast, the analysis of the mRNA level quantified for the *FOXO3* gene (Figure 7E) showed its increase in all experimental groups as compared to CTR. In COCs subjected to 3D-IVM in the presence of Vnz, this increase was 7 times higher (6.8 ± 2.34) than in CTR. The difference was statistically significant (*p* < 0.001). In 3D-IVM-generated COCs treated with Ndn, the determined mRNA level was 5 times higher (4.4 ± 0.23) than in CTR, and the difference between these values turned out to be statistically significant (*p* < 0.01). In COCs undergoing exposure to CsA (3.0 ± 1.00) during 3D-IVM, the observed increase in the quantitative profile of *FOXO3* transcripts was statistically insignificant (*p* = 0.17).

#### 2.5.3. Quantitative Analysis of Intracytoplasmic Glutathione Concentration in 3D-IVM-Generated COCs Treated with the Selected Endocrine Disruptors

**Figure 8:**
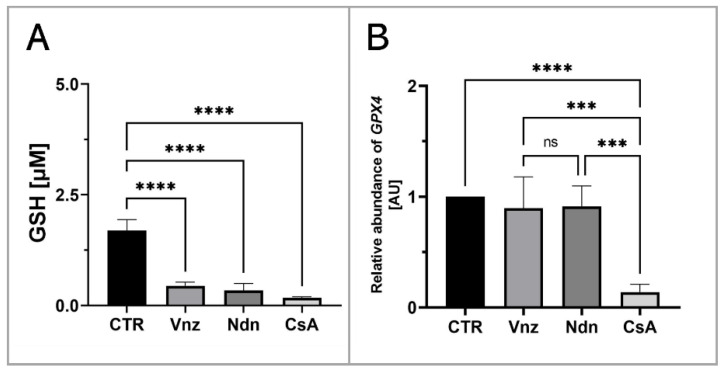
Quantitative analysis of intracytoplasmic glutathione concentration (**A**) and *GPX4* gene (**B**) shown by RT-qPCR at the transcript level in porcine COCs subjected to the IVM procedure using a 3D culture model (alginate capsules) in control cultures (CTR) or in experimental cultures: in the presence of vinclozolin (Vnz), nandrolone (Ndn) or cyclosporin A (CsA). The results are presented as mean values with *n* = 5 ± standard deviation (SD), and each “*n*” consisted of 50 COCs. Statistical analysis: homogeneity of variance—Brown–Forsythe test; normality of distribution—Bartlett’s test and one-way ANOVA followed by Dunnett’s post hoc test, *** *p* < 0.001, **** *p* < 0.0001; ns—nonsignificant.

To assess the influence of the selected EACs on the COCs’ intracytoplasmic concentration of glutathione (GSH), its abundance in the isolated total protein was measured. Based on the obtained results, a decrease in the GSH intracytoplasmic concentration was recognized in all experimental groups as compared to the level of 1.7 ± 0.24 μM/mL estimated for the CTR group. In COCs subjected to 3D-IVM in the presence of Vnz, Ndn and CsA, the GSH concentrations reached levels as follows: 0.4 ± 0.09 μM/mL, 0.3 ± 0.16 μM/mL and 0.2 ± 0.02 μM/mL. The differences between the experimental and CTR groups were found to be statistically significant (*p* < 0.0001) (Figure 8A).

Additionally, to examine whether the observed decrease in the intracytoplasmic concentration of GSH might be a result of the intensive process of eliminating ROS, the rate of RA was estimated for mRNA transcribed from the *GPX4* gene encoding glutathione peroxidase 4, which is responsible for the reduction of, among others, lipid peroxides. Ascertaining the quantitative profile of *GPX4* transcripts confirmed their almost 10-fold decrease (0.1 ± 0.07) in 3D-IVM-derived COCs exposed to CsA as compared to CTR. This diminishment turned out to be statistically significant (*p* < 0.0001). No statistically significant changes (*p* = 0.83 and *p* = 0.9) in the levels of mRNA transcripts identified for the *GPX4* gene were shown in the subpopulations of COCs undergoing 3D-IVM in the presence of Vnz (0.9 ± 0.28) or Ndn (0.9 ± 0.18) (Figure 8B).

### 2.6. Analyzing the Extent of Meiosis/Maturation Progression in Oocytes Treated with the Selected Endocrine Disruptors during IVM Procedure under 3D Culture Conditions

**Figure 9:**
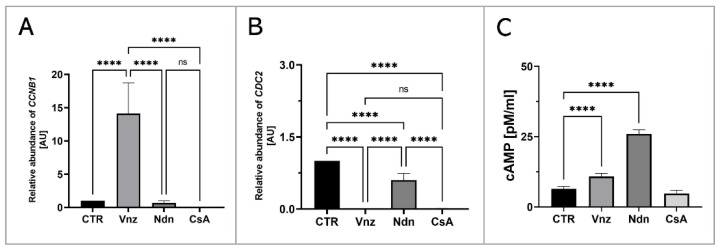
Analysis of the extent of meiosis/maturation progression in porcine COCs subjected to the IVM procedure using a 3D culture model (alginate capsules) in control cultures (CTR) or in experimental cultures: in the presence of vinclozolin (Vnz), nandrolone (Ndn) or cyclosporin A (CsA). (**A**,**B**): Quantitative profiles estimated for expression of *CCNB1* and *CDC2* genes, shown by RT-qPCR at the transcript level. The results are presented as mean values with *n* = 5 ± standard deviation (SD), and each “*n*” consisted of 50 COCs. (**C**): Analysis of intracytoplasmic cAMP concentration. Statistical analysis: homogeneity of variance—Brown–Forsythe test; normality of distribution—Bartlett’s test and one-way ANOVA followed by Dunnett’s post hoc test, **** *p* < 0.0001; ns—nonsignificant.

In order to assess the influence of the selected EACs on the oocyte maturation process, the RAs of mRNA molecules that were biosynthesized as a result of transcriptional activities of genes encoding regulatory and biocatalytic subunits of a heterodimeric meiosis/maturation-promoting factor, designated as *CCNB1* (cyclin B1) and *CDC2* (cyclin-dependent kinase 1), were ascertained. The quantification of mRNA expression for the *CCNB1* gene showed its almost 15-fold increase in COCs subjected to 3D-IVM in the presence of Vnz (14.1 ± 4.59) as compared to the CTR group. This intergroup variability was statistically significant (*p* < 0.0001). In turn, no statistically significant changes (*p* = 0.99 and *p* = 0.93) within the quantitative profiles of mRNA transcripts identified for the *CCNB1* gene were observed in 3D-IVM-derived COCs exposed to Ndn (0.7 ± 0.31) or CsA (undetectable) (Figure 9A). Interestingly, quantitatively analyzing the RA of *CDC2* mRNAs revealed their significant decrease in all experimental groups (Vnz = 0.002 ± 0.002, Ndn = 0.6 ± 0.14, CsA = 0.012 ± 0.003) as compared to CTR (*p* < 0.0001) (Figure 9B).

Additionally, to assess the effect of the selected EACs on the level of 3′,5′-cyclic AMP (cAMP), its concentration in the isolated total protein was determined using protein extracts stemming from the 3D-IVM-generated COCs (Figure 9C). Based on the obtained results, the highest concentration of cAMP of 26 ± 1.43 pM/mL was found in 3D-IVM-derived COCs cultured in the presence of Ndn. There was a statistically significant difference (*p* < 0.0001) in the measured cAMP concentrations between Ndn-treated and untreated (6.4 ± 0.88 pM/mL) subpopulations of COCs undergoing 3D-IVM. In COCs cultured in the 3D-IVM model and simultaneously treated with Vnz, a cAMP concentration of 10.8 ± 1.12 pM/mL was estimated. The difference in the measured cAMP levels between Vnz and CTR groups was statistically significant (*p* < 0.0001). Exposure of porcine COCs to CsA during 3D-IVM did not cause the occurrence of statistically significant changes (*p* = 0.054) in the level of cAMP (4.7 ± 1.12 pM/mL) as compared to CsA-unexposed subpopulations of COCs.

## 3. Discussion

So far, the direct consequences of long-term exposure to the EACs/EDCs tested in the current study, which might lead to the disturbed regulation of mammalian oocyte maturation, have not been precisely recognized. The research undertaken by our team allowed us to obtain completely new information about the possible negative impacts of the selected endocrine disruptors (Vnz, Ndn and CsA) on the quality of oocytes, which determines the reproductive success of females dependent on efficiently terminating meiotic maturation under not only in vivo physiological conditions but also ex vivo conditions associated with experimental and applied embryology and ART strategies, including the IVM procedure. The concentration of Vnz in the aquatic environment obviously directly influences the fish living there. This is especially true of the disrupted development of their reproductive system. Taking this finding into consideration, decreased egg production and progressive degeneration of oocytes have been found in females of the *Pimephales promelas* species [93]. In turn, in *Oryzias latipes*, the termination of oogenesis at its early stages (i.e., pre-vitellogenesis and initial stages of vitellogenesis) was proven by the results of investigations by Kiparissis et al. [5]. As a consequence of the increased concentration of Vnz in the aquatic environment, both augmented concentration of testosterone in blood plasma and a reduction in ovarian weight in relation to the total body weight in adult *Pimephales promelas* females were shown by Makynen et al. [94]. Of particular interest, an increased bioconcentration of this fungicide in the tissues of females was observed compared to males of this species, which may be related to the different contents of lipids in the two sexes. In mammals, the influence of Vnz on the development and functioning of the female and male reproductive systems has also been confirmed. For example, administration of Vnz to pregnant female rats triggered developmental disorders of the reproductive system in male and female embryos, which was a transgenerational effect. In male rats, an increased incidence of apoptosis in spermatogonia, together with lower sperm counts and diminished motility, was identified [95]. On the other hand, in female rats, Vnz determined the course of pregnancy in subsequent generations (anemia of pregnant animals and intrauterine hemorrhage). There was also progressive feminization of male embryos and masculinization of female ones [96]. As for the direct negative impact of Vnz on the human body, a negative correlation has been demonstrated between the high concentrations of pesticides, including Vnz (3000 g/ha of arable land) in surface waters, and the reduced quality of oocytes in women living in the northern part of France in Picardy [97].

Our present investigations carried out on 3D-IVM-derived COCs treated with Vnz show, for the first time, that Vnz induces the apoptosis of cumulus cells by enhancing the expression of p53, Bad and caspase 3 proteins, which gives rise to the internucleosomal biodestruction of the nuclear genome. These findings are supported by observations obtained previously with the use of whole porcine follicles exposed to Vnz [10]. The p53 protein directly activates the transcription of the *FOXO3* gene in response to DNA damage and brings about the promotion and progression of apoptotic cell death [98]. This is consistent with the results achieved in our study, which were confirmed by the increased RA of *FOXO3* mRNA in COCs maturing in the presence of Vnz. In turn, FOXO3 is responsible for the induction of apoptosis through interactions with the BIM protein, which is associated with the mitochondrial pathway of apoptosis [99]. Research undertaken by Obexer et al. [100] and Hagenbuchner et al. [101] demonstrated that intramitochondrial accumulation of ROS in human neuroblastoma cells contributes to the fusion of FOXO3 with the BIM protein, which, in turn, evokes mPTP opening and subsequently cytochrome c leakage. This mechanism also appears to be prompted in response to the action of Vnz in CCs. The intensified occurrence of late-apoptotic symptoms in CCs correlates with a decreased fertilization rate and, consequently, attenuated quality of human IVF-derived embryos [102]. The use of protein microarrays showed a significant increase in the expression of the livin protein, which belongs to the family of proteins that inhibit apoptosis [103]. Its action is based on blocking the extrinsic apoptotic pathway, regulated by the Fas/FasL pathway [104] and TNF-β [103]. The p53 protein, which imitates the function of a transcription factor for the receptors involved, is also engaged in the activation of this pathway [105]. Enhanced expression of livin in 3D-IVM-generated COCs exposed to Vnz, which was identified in our current study, may indicate the onset of cell defense mechanisms against apoptosis caused by the action of this fungicide.

The analysis aimed at examining the influence of Vnz on mitochondria revealed that this endocrine disruptor induced the augmentation of the quantitative profile of mRNA transcribed from the *ATP5A1* gene encoding the ATP synthase subunit. This result is also reflected in the level of ATP produced and in the intensity of MtOR staining. The significant increase in the level of proton leak seems to be interesting, which indicates damage to the mitochondrial membrane [106] and is associated with the generation of ROS [107]. The loss of integrity within the ultrastructure of the mitochondrial membrane leads to a decrease in the membrane potential and, consequently, to the dwindling of ATP levels [108]. Elevated ROS concentration is one of the factors that promote the necrosis process in murine embryonic fibroblasts [109,110], and as has been shown in our previous studies [11], Vnz can also induce pro-necrotic alterations in granulosa cells in porcine ovarian follicles. Additionally, a high level of ROS in the oocyte initiates germinal vesicle breakdown (GVBD); however, the extrusion of the polar body into perivitelline space is blocked [111]. Immediately before GVBD, there is a sudden increase in ATP levels [112], which was observed during the Seahorse XFp analysis. On the other hand, an elevated concentration of cyclin B accelerates the disintegration of the germinal vesicle due to premature activation of MPF [113]. All of these findings provide strong evidence that treatment of porcine COCs with Vnz results in a considerable diminishment of their molecular quality by promoting proapoptotic changes in CCs and expediting GVBD processes within oocytes.

A panel of the presented studies focused on the 3D-IVM of COCs undergoing exposure to Ndn indicated the rapid increase in the intraooplasmic cAMP concentration. Perpetuation of a high intracytosolic concentration of cAMP is a pivotal biomarker of the inhibition of the process of nuclear maturation of the oocyte, which arises from blocking the resumption of meiosis I [114,115]. The analysis of the oocyte ultrastructure revealed the accumulation of large (˃3 μm) lipid droplets (LDs) in the oocyte, which are irregularly distributed in the cytoplasm. Even though the presence of many LDs is one of the factors determining the increased developmental competence of the oocyte [70,116], the occurrence of intracytoplasmic lipid droplets exhibiting such morphology and distribution is characteristic of immature oocytes [117]. The extensive and nonspecific storage of lipid droplets in the cytoplasm of oocytes subjected to 3D-IVM in the presence of Ndn could explain the observed low level of ATP synthesis in COCs. Taking into account the enhanced intraooplasmic accumulation of LDs and the corresponding decline in mitochondrial activity that were proven in our current investigation, the results of the studies by He et al. [118], in which LD accumulation in response to intramitochondrial metabolic quiescence was identified, seem to be particularly interesting. Moreover, oocytes grown in melatonin-supplemented medium also displayed intracytoplasmic accumulation of LDs at one pole [118]. The authors of the research undertaken to empirically explore the molecular nature of human hepatocarcinoma-derived cell lines (HepG2) hypothesized that nandrolone, by inhibiting complex III (i.e., cytochrome complex) in the respiratory/electron transport chain, brings about the promotion of intracellular antioxidative events. These events give rise to the rapid elimination of ROS in HepG2 cells [34]. In turn, the drastically dwindling of the concentration of ROS that arises from their scavenging promotes the dormancy of healthy or cancerous stem cells [34]. For that reason, additional extensive studies should be performed to confirm whether such a mechanism is activated in oocytes during their IVM. Moreover, it appears to be of great importance to examine the relationship between β-oxidation of fatty acids and glucose metabolism in COCs.

A panel of TUNEL-based investigations demonstrated that the highest percentage of late-apoptotic cumulus cells occurred in COCs subjected to 3D-IVM in the presence of nandrolone. The results obtained with the use of protein microarrays confirmed the proapoptotic effect of Ndn. Ndn has been shown to enhance the expression of caspase 3 at both mRNA and protein levels. These findings are consistent with those achieved in the study by Bordbar et al. [119], in which the induction of apoptosis in granulosa cells stemming from primary follicles was observed in rats treated with Ndn. Additionally, exposure to Ndn was found to decrease blood levels of LH, FSH, estrogens and progesterone. Moreover, the protein microarray-mediated analysis revealed that the expression of cytochrome c increases rapidly in COCs cultured in the 3D-IVM model associated with Ndn treatment. Thus, long exposure of COCs to Ndn triggers damage to the mitochondria. Therefore, the level of ATP synthesis is sharply reduced, as was evidenced by the declined RA of mRNA transcripts synthesized from the *ATP5A1* gene coding for the ATP synthase subunit. A similar negative effect of Ndn on ATP synthesis was recognized in HepG2 cells and nerve cells in the research by Agriesti et al. [34] and Carteri et al. [120], respectively. In contrast, other investigators discovered the existence of a positive correlation between increased ATP levels and high in vitro developmental competence of bovine and human oocytes [121,122]. In turn, biodegradation of mitochondria, elevated ROS concentration and diminished ATP production accelerate follicular atresia and contribute to the premature extinction of ovarian function in women under the age of 40 years [123].

Especially interesting results achieved within the framework of the present exploration are those identifying an increase in the expression of TNF-β (α-lymphotoxin) and HSP27 proteins in COCs undergoing 3D-IVM and simultaneous exposure to Ndn. The cell synthesizes HSP27 in response to stressors triggering apoptosis [124], as was also supported by the results of clinical studies focused on female patients with diagnosed ovarian cancer [125]. On the other hand, the activation of TNF-β-related signaling pathways can lead to neoplastic cell transformation in liver and prostate cancer development [126]. The histological compartment of granulosa cells within the ovarian follicle represents a cell subpopulation that is characterized by high plasticity [127,128]. Its effect is not only the differentiation of cells within the granulosa layer into mural cells, antral cells and the cells forming the cumulus oophorus but also their involvement in the formation of the corpus luteum [129]. Furthermore, human granulosa cells have been shown to display certain attributes specific to stem cells [128,130] and can exhibit capabilities to differentiate into neurons, osteocytes, chondrocytes, hepatocytes and adipocytes under appropriate in vitro culture conditions [127,131,132,133]. Moreover, studies conducted on granulosa cells isolated from porcine ovarian follicles demonstrated their differentiability into osteoblasts and fibroblasts [134]. In turn, bovine granulosa cells were successfully transformed into endothelial cells under in vitro culture conditions [135]. All of the aforementioned findings provide clear evidence for the enormous plasticity of granulosa cells, which also allows them to undergo neoplastic transformation [136]. One of the types that represent highly malignant and metastatic neoplasms originating from granulosa cells is ovarian granulosa-cell tumor (folliculoma) [137,138]. Therefore, taking into consideration not only the above-mentioned data justifying the potential of granulosa cells for cancer formation but also our previous investigation [35] and the results of the present research (indicating elevated expression of TNF-β and HSP27 in the presence of Ndn), it can be concluded that Ndn enhances the risk of neoplastic transformation of CCs derived from COCs subjected to IVM in a microenvironment enriched with the presence of this EDC. This scientific judgment is consistent with the results of studies by Agriesti et al. [34], who convincingly verified that Ndn increases the risk of oncogenic transformation in the cells by changing their phenotype to a stem cell-like phenotype.

CsA is a frequently used immunosuppressant [139], and due to its protective effect, it is also a factor applicable in studies focusing on the exploration of mitochondrial function [140]. A side effect of the use of CsA is a decrease in the level of GSH synthesis, which, in turn, may increase the concentration of ROS in cells [141]. Previous studies have shown that CsA induces the processes of autophagy (self-digestion) in ex vivo expanded human tubular cells and also under in vivo conditions in the kidneys of rats. This is most likely a protective mechanism against the cytotoxicity of CsA [142,143,144]. The factor that stimulates autophagy is unfolded proteins, which are responsible for the activation of proteostatic stress-related events in the cisternae and tubules of the endoplasmic reticulum [145,146]. Conversely, long-term stress can induce apoptosis in a model of chronic nephropathy [147]. In our current investigation, this differential response to CsA was also identified in CCs during the IVM of porcine COCs. Protein analysis of apoptotic pathways revealed increased expression of executioner caspase 3, and RT-qPCR analysis revealed the highest level of *LC3* mRNA in COCs undergoing 3D-IVM in the presence of CsA. The intense autophagy occurring in oocytes subjected to 3D-IVM in the CsA-enriched medium was recognized by the intracellular abundance of autophagosomes observed during TEM analysis of COCs. However, despite the autophagy and apoptosis taking place in CCs, oocyte maturation was not inhibited, as was evidenced by not only the smaller number of large lipid droplets, whose diameter ranged from 1 to 3 μm, but also their perinuclear location, which is a characteristic feature of meiotically matured (i.e., MII-stage) oocytes [117].

Although CsA has been previously found to exert a protective effect on mitochondria [46,47], the detection of mitophagy in CCs originating from COCs subjected to 3D-IVM in the CsA-enriched medium implies that this endocrine disruptor prompted mitochondrial damage. In turn, the damaged mitochondria detrimentally affect intracellular metabolism by promoting the production of ROS and ultimately triggering apoptosis [148,149]. The harmful impact of CsA on the mitochondria within CCs is confirmed by the dwindling of ATP synthesis, which was identified after 3D-IVM of COCs in the presence of CsA. A similar effect was observed in breast cancer cells [150]. In addition, CsA not only reduced the mitochondrial membrane potential (ΔΨm) that is generated by proton pumps (complexes I, III and IV) in the process of energy storage (i.e., respiratory biosynthesis of ATP) during oxidative phosphorylation but also triggered the enhancement of caspase 3 activity [151,152]. On the one hand, mitochondrial transmembrane potential is a key indicator of mitochondrial metabolic activity, because it reflects the process of electron transport and oxidative phosphorylation, the driving force behind ATP production. On the other hand, ΔΨm represents an intermediate energy store used by ATP synthase to accumulate ATP [153]. Therefore, based on the obtained results, it can be concluded that although CsA does not interfere with the nuclear and cytoplasmic maturation of the oocyte, it may decrease the molecular quality of oocytes by diminishing ATP biosynthesis in COCs [154,155]. A similar effect that gave rise to the attenuation of the quality of oocytes due to low ATP levels was observed by Groth et al. [48] in a murine model study using CsA. These investigators demonstrated that exposure of female mice to high doses of CsA (at levels oscillating between 20 and 30 mg/kg) contributed to lessening the incidence of embryo implantation and postimplantation survival rates of conceptuses [48]. The reason for such changes was a remarkable decline in the quality of oocytes, which did not display the sufficiently abundant bioenergetic reservoirs required for the proper development of the embryo and deposited/accumulated in the intramitochondrial ATP biomachinery located within oxysomes (also known as F0-F1 particles) inside the folds of the cristae in the inner mitochondrial membrane.

One of the quite new applications of CsA is anticancer therapy for non-small cell lung cancer (NSCLC), which accounts for approximately 85–90% of all lung cancer diagnoses. In phase I/II clinical trials, CsA was administered to patients in two doses: low (1–2 mg/kg daily) and higher (3–6 mg/kg daily). Administration of a low dose has been found to increase the chance of survival by two years [156]. In turn, research undertaken by Qin and Chen [157] to explore the molecular nature of the A549 non-small cell lung cancer cell line discovered that CsA augments the proliferative activity and metabolism of glucose in neoplastic cells. In addition, the elevated lipid accumulation that was evidenced in A549 non-small cell lung cancer cells brought about the enhanced production of ROS prompted by CsA action. The aforementioned authors hypothesized that the pleiotropic effect of CsA is dependent on cell metabolism, and its use may induce neoplastic changes in patients after organ transplantation [157]. The potential neoplastic activity may be demonstrated by the enhanced expression of TNF-β, which leads to the activation of pathways related to neoplastic transformation [126]. On the other hand, studies aimed at recognizing tumorigenic scenarios specific to prostate cancer cells have resulted in determining the predominant role of CsA both in attenuating/alleviating the capability of neoplastic cells to migrate/metastasize and in suppressing tumor growth, which can be directly elucidated by CsA-induced intensification of apoptosis occurrence [158]. Collectively, the results of a panel of studies aimed at examining the influence of CsA on oocytes undergoing 3D-IVM in its presence led to the conclusion that although CsA may stimulate cytoplasmic and nuclear maturation of the oocyte, at the same time, by reducing ATP levels, it may diminish the molecular quality of oocytes after the completion of the IVM procedure.

To sum up, recognizing the molecular mechanisms by which a triad of EACs/EDCs (Vnz, Ndn and CsA) trigger disturbances in the intracellular bioenergetic balance dependent on mitochondrial compartments is crucial for achieving satisfactory outcomes related to the extracorporeal maturation of porcine oocytes. Generating a sufficiently high percentage of porcine IVM-derived oocytes whose molecular quality has not been attenuated by EDC-prompted impairments in intramitochondrial bioenergetic homeostasis is greatly important from multiple points of view. One of them is the ability to perpetuate such availability of ex vivo–matured oocytes that might ensure abundant sources of not only ova highly capable of being fertilized by conventional IVF or ICSI but also excellent-quality nuclear recipient cells for SCNT-based cloning in pigs. The above-mentioned requirements could be fulfilled by developing the new strategy of 3D-IVM, which allowed us to improve the efficiency of the ex vivo maturation of porcine oocytes in a culture microenvironment deprived of EACs/EDCs.

The improvement of parameters related to molecular quality in recipient oocytes that have been matured in vitro under 3D culture conditions and have subsequently received donor somatic cell nuclei (DSCN) can result, among others, from retaining largely balanced biogenesis, reduplication, cytophysiological functions and distribution patterns observed for reservoirs of intraooplasmic mitochondrial compartments. The maintenance of this dynamic balance seems to be due to a remarkably diminished incidence of mitophagic and apoptotic events, which, in turn, can lead to a lack or only negligible occurrence of dysregulations in sustainable intramitochondrial ATP biosynthesis. For all of the aforementioned reasons, SCNT-mediated reconstruction of porcine enucleated oocytes that display augmented molecular quality might give rise to generating cloned embryos, in the blastomeres of which faithful and synergistic intergenomic crosstalk between nuclear and mitochondrial DNA fractions takes place. The scenario of successful transcriptional and proteomic communication between nuclear and mitochondrial compartments is undoubtedly responsible for enhancing the capabilities of DSCN to be epigenetically reprogrammed in SCNT-derived pig embryos stemming from high-quality recipient oocytes.

Furthermore, our current research contributed to successfully devising ex vivo models based on 3D-IVM of porcine oocytes undergoing treatments with a variety of EACs/EDCs (Vnz, Ndn or CsA), which might also provide promising and hopeful biomedical tools for the establishment of experimental, preclinical and clinical therapies aimed at the next-generation nonsurgical treatment modalities of acquired subfertility and fertility cases diagnosed in human female patients. In such sub-fertile or infertile women, ovarian follicles can be characterized by symptomatic failures in the in vivo maturation of oocytes due to overexposure of their reproductive systems to the selected ectopic/environmental EDCs (Vnz, Ndn and CsA), followed by the overabundant accumulation of this triad of endocrine disruptors in different ovarian tissue compartments.

Finally, in light of the results achieved in our present investigation, which suggest that Ndn not only reduces the in vitro developmental competence of pig oocytes by disturbing the metabolism of CCs, but also increases the incidence of cancerous conversion (tumorigenesis) in the cumulus oophorus compartment of porcine COCs cultured in the novel model of 3D-IVM, it is particularly important to educate young people about the risk and dangerous side effects arising from misuse of this AAS. It is worth highlighting that, according to data presented in 2021 by the Addiction Center, 1 in 50 students aged 17–18 used steroids. In turn, according to the Food and Drug Administration data, 375,000 boys and 175,000 girls abuse AASs annually (https://www.addictioncenter.com/stimulants/steroids/, accessed on 1 January 2008). The main motivation for taking AASs is the desire to improve one’s own appearance in a short time, without physical exertion. For those reasons, developing new-generation ex vivo models designed using 3D-IVM-mediated culture systems of porcine COCs that have been subjected to single or combined overexposure to Vnz, Ndn and CsA might be a reliable and feasible approach to developing and optimizing anticancer treatment modalities within the framework of targeted antioncogenic therapeutics dedicated to female patients afflicted with ovarian follicle-specific oncologic diseases. The etiopathogenesis of these diseases can be related to tumorigenic transformation of cumulus oophorus-based cytological compartments stemming from EAC/EDC-overexposed ovaries into malignant and metastatic neoplasms.

## 4. Materials and Methods

### 4.1. Collection and In Vitro Maturation of Porcine Cumulus–Oocyte Complexes under 3D Culture Conditions

Ovaries that displayed the absence of corpora lutea were collected from ~4–5-month-old (i.e., prepubertal) gilts at a local abattoir under veterinarian control within 20 min of slaughter. For isolation of COCs, approximately twenty ovaries from ten female pigs were collected for each experiment. Ovaries were transported to the laboratory in sterile phosphate-buffered saline (PBS; pH 7.4, 38 °C, PAA The Cell Culture Company, Piscataway, NJ, USA) containing 1% (*v*/*v*) antibiotic/antimycotic solution (AASol; Thermo Fisher Scientific, Waltham, MA, USA) within ~1 h. Afterwards, ovaries were rinsed twice with sterile PBS and transferred to handling medium (HM). The latter comprised Tissue Culture Medium 199 (TCM 199; Sigma-Aldrich, St. Louis, MO, USA) supplemented with 10% (*v*/*v*) fetal bovine serum (FBS; Thermo Fisher Scientific) and 1% (*v*/*v*) AASol at a temperature of 38 °C. COCs were aspirated from morphologically normal medium-sized follicles (4–6 mm in diameter) using 28G needle having a size of 5/8” attached to a disposable syringe. For each experiment, a total of 100 COCs, which exhibited a homogenous ooplasm, and 3 to 4 compact layers of cumulus cells were selected for in vitro maturation (IVM) [49]. The procedure of IVM was performed according to the technique described by Pedersen et al. [159]. The group of COCs intended for IVM were encapsulated in fibrin-alginate hydrogel beads (FABs) according to a previously reported protocol [160]. In detail, a subpopulation ranging from 3 to 5 COCs along with a minimum volume (up to 5 μL) of maturation medium were precisely transferred (with the use of micropipette) into a drop of mixture of fibrinogen and alginate (FA: 0.5% alginate solution and 50 mg/mL fibrinogen solution mixed in a 1:1 ratio; Sigma-Aldrich). The maturation medium (MM) consisted of TCM 199 medium enriched with 10% (*v*/*v*) FBS, 1% (*v*/*v*) AAS, 10 IU/mL pregnant mare serum gonadotropin (PMSG; RayBiotech Life, Inc. Peachtree Corners, GA, USA), 5 IU/mL human chorionic gonadotrophin (hCG; Sigma-Aldrich), 0.004 mg/mL L-glutamine (Sigma-Aldrich) and 10% porcine follicular fluid (pFF). Subsequently, 7.5 μL volumes of thrombin/Ca^2+^ solution (Sigma-Aldrich) was pipetted on each FA drop. The occurring FABs were transferred to a 5% CO_2_ incubator (38 °C, 5 to 7 min) using “incubation chambers” on the Petri dish. After this time, simultaneous gelation of fibrin and alginate was observed. In the next step, FABs with COCs inside were transferred to 96-well plates (one capsule per well, Nunc™, Thermo Fisher Scientific) containing 100 μL of MM. After 24 h of preculture, COCs were randomly allotted to a control group (CTR) and three experimental groups. For the first, second and third experimental groups, the endocrine-disrupting chemicals (EDCs) vinclozolin (Vnz; at a concentration of 10^−7^ M/mL, Sigma-Aldrich), nandrolone (Ndn; at a concentration of 10^−5^ M/mL, Sigma-Aldrich) and cyclosporine A (CsA; at a concentration of 1 µM/mL, Sigma-Aldrich) were added to the MM, respectively. The experimental doses of the above-mentioned EDC agents used were established on the basis of literature reports and our own research [9,35,158]. Cultures were carried out for a further 48 h at 38 °C in an atmosphere of 5% CO_2_ and 95% relative humidity. Following completion of IVM procedure, the maturation status of in vitro cultured COCs was initially assessed by observing them under an inverted microscope at a magnification ranging from 10× to 60× (Nikon Ti-U microscope equipped with a Nikon DS-Fi1c-U3 camera; Tokyo, Japan). The assessment of in vitro–matured COCs was accomplished not only by identifying the extrusion of the first polar body (PB) into perivitelline space but also by evaluating the extent of dispersion/expansion of CC layers surrounding each oocyte. The microscopic diagnostics of the aforementioned morphological biomarkers specific for ex vivo–matured COCs were also supported by analyzing molecular biomarkers of meiotic maturity of oocytes encompassing highly enhanced expression profiles for genes encoding MPF and the strongly reduced levels of cAMP, as described in Section 4.3 and Section 4.6, respectively.

### 4.2. Total RNA Isolation and cDNA Synthesis

Total RNA was extracted from both COCs undergoing 3D-IVM in the presence of selected EACs and COCs cultured without addition of Vnz, Ndn or CsA. Total cellular RNA was isolated using the EZ-10 Spin Column Total RNA Mini Preps Super Kit (Bio Basic Canada Inc.; Markham, ON, Canada) according to the manufacturer’s protocol. The quantity and quality of the total RNA were ascertained by measuring the absorbance at detection wavelengths λ equal to 260 nm and 280 nm with a NanoDrop ND2000 Spectrophotometer (Thermo Fisher Scientific). Moreover, RNA samples were electrophoresed on a 1% (*w*/*v*) denaturing agarose gel to verify the RNA quality and stored frozen at −80 °C. First-strand cDNA was prepared by reverse transcription (RT) using 1 mg of total RNA, random primers and a High-Capacity cDNA Reverse Transcription Kit (Applied Biosystems; Foster City, CA, USA) according to the manufacturer’s protocol. The 20 mL total reaction volume contained random primers, dNTP mix, RNAse inhibitor and Multi Scribe Reverse Transcriptase. RT was performed in a Veriti Thermal Cycler (Applied Biosystems; Foster City, CA, USA) according to the following thermal profile: (1) 25 °C for 10 min, (2) 37 °C for 120 min and (3) 85 °C for 5 min. Genomic DNA amplification contamination was checked using control experiments, in which reverse transcriptase was omitted during the RT step. The samples were kept at −20 °C until further analysis.

### 4.3. Quantitative Reverse Transcriptase Real-Time Polymerase Chain Reaction (qRT-PCR)

RT-qPCR was performed according to the manufacturer’s protocol. To quantitatively assess the transcriptional activities identified for each analyzed gene, the RT-qPCR reactions were successfully initiated and subsequently completed for each sample using a reaction mix prepared as follows: 1 × SYBR Select Master Mix (Thermo Fisher Scientific), 2 μL of forward and reverse primers (1 μM each) and 4 μL of 20 × diluted cDNA in a final volume of 15 μL. No-RT control run was conducted with DNase-digested RNA to verify that the digestion was successful and sufficient for selected samples. The amplification protocol included an initial preheating at 50 °C for 2 min, initial denaturation at 95 °C for 2 min and 40 cycles of amplification (15 s at 95 °C and 60 s at 60 °C). A melting curve analysis was performed at the end of each run. RT-qPCR was carried out with a Rotor-Gene Q (Qiagen, Hilden, Germany). The sequences of all RT-qPCR primers are presented in Table 1.

Alterations in the quantitative profiles (i.e., relative abundances; RAs) of relevant mRNA transcripts that were triggered by the exposure of in vitro maturing COCs to the selected EACs were rendered as the ratio of the target gene versus the reference *GAPDH* gene (coding for glyceraldehyde-3-phosphate dehydrogenase) in relation to expression in control samples using the method developed and optimized by Pfaffl [166] according to the following equation:

$$\mathrm{Ratio}=\frac{{\left(E_{\mathrm{target}}\right)}^{\mathrm{nCt}}{\mathrm{Target}}^{\left(\mathrm{control}-\mathrm{sample}\right)}}{{\left(E_{\mathrm{reference}}\right)}^{\mathrm{nCt}}{\;\mathrm{Reference}}^{\left(\mathrm{control}-\mathrm{sample}\right)}}$$

In the above-indicated algorithmic formulation, the mathematical designation E denotes the amplification efficiency, whereas the Ct symbol is assigned to the number of RT-qPCR cycles needed for the signal to exceed a predetermined threshold value.

### 4.4. The Use of Apoptosis Proteome Profiler Arrays for Detailed Evaluation of Pro- and Antiapoptotic Pathways in 3D-IVM-Derived COCs Treated with the Selected Endocrine Disruptors

Analysis of the molecular mechanism that underlies the proteomic networks related to either initiation and progression or inhibition of programmed cell death in EDC-treated COCs undergoing 3D-IVM was performed using the RayBio^®^ Human Apoptosis Antibody Array Kit (RayBiotech, Inc., Norcross, GA, USA) according to previously reported protocol [10] with additional modification. This technique detects 43 proapoptotic and antiapoptotic proteins involved in promoting or suppressing the process of programmed cell death. The COCs were washed in sterile PBS. Total protein was extracted using the cell lysis buffer contained within the kit. After lysis by freeze–thawing and subsequent homogenization, lysates were centrifuged at 14,000 rpm for 10 min at 4 °C, and supernatants were stored at −70 °C until further analyses. The protein concentration of each sample was quantified using the NanoDrop ND2000 Spectrophotometer (Thermo Fisher Scientific). Protein array membranes were immersed in the blocking buffer and incubated with 600 mg of proteins overnight at 4 °C. After washing with the kit buffers, samples were incubated overnight with biotinylated detection antibodies at 4 °C, and after further washing, they were exposed to Alexa Fluor 555 dye-conjugated streptavidin overnight. Signals in the array membranes were detected and quantitated on a ChemiDoc chemiluminescence imaging system (Bio-Rad Laboratories Inc., GmbH, Munchen, Germany). For each protein signal, absorbance was determined using the Antibody Array Analysis Tool (RayBiotech Life, Inc., Peachtree Corners, GA, USA). Intensities of individual spots were normalized to an internal positive control (standardized amounts of biotinylated immunoglobulin G) printed directly onto the array, according to an algorithm specified by the manufacturer. The measurement for each protein was repeated twice in two independent trials (*n* = 5 for each protein in one experimental group) in each of the experimental groups. From the obtained values for each of the detected proteins, the mean value and standard deviation were obtained for each experimental group. Only the expression data of proteins that are directly involved in apoptosis and had a statistically significant increase after treatment are shown.

### 4.5. TUNEL-Assisted Detection of Late-Apoptotic Cells in COCs Undergoing Exposure to the Selected EACs during 3D-IVM

The In Situ Cell Death Detection Kit (Roche, Mannheim, Germany) was used to perform a terminal deoxynucleotidyl transferase (TdT)-mediated 2′-deoxyuridine-5′-triphosphate (dUTP) nick-end labeling (TUNEL) assay that was dependent on fluorescein-5-isothiocyanate (FITC) tags conjugated with dUTP nucleotides. The TUNEL assay provides a method that enables us to identify and ascertain the extent of internucleosomal DNA fragmentation arising due to the progression of apoptotic cell death to its final destructive phase. After terminating the 3D-IVM of COCs, the MM was removed from plates, and COCs were rinsed twice with sterile Ca^2+^ -and Mg^2+^-depleted PBS (PAA The Cell Culture Company). In the next step, COCs were incubated in fixation solution composed of 4% paraformaldehyde (PFA; Santa Cruz Biotechnology Inc. Dallas, TX, USA) in PBS for 1 h at room temperature. Then, COCs were subjected to three-step washing with PBS (each step for 10 min) followed by permeabilization in 0.1% sodium citrate (Sigma-Aldrich) for 2 min on ice. Afterwards, TUNEL assay was performed according to the previously reported protocol [167]. Firstly, the COCs were treated with proteinase K (Promega Corporation, Madison, WI, USA) in a humidity chamber for 15 min at 37 °C. Secondly, COCs were rinsed twice with PBS and subsequently exposed to 3% hydrogen peroxide (H_2_O_2_; Sigma-Aldrich) in methanol for 10 min to block endogenous peroxidase activity. Thirdly, COCs were washed twice with PBS and deposited into the medium supplemented with 5% bovine serum albumin (fraction V; BSA-V, Sigma-Aldrich) for 20 min to block nonspecific binding. The COCs were then rinsed twice with PBS, followed by treatment with TUNEL mixture composed of TdT and FITC-tagged dUTP in a humidity chamber for 1 h at 37 °C in the dark. After the TUNEL reaction was completed, the COCs were washed thrice with PBS and counterstained with 4′,6-diamidino-2-phenylindole (DAPI) diluted in VECTASHIELD Antifade Mounting Medium (Vector Laboratories, Burlingame, CA, USA). Finally, the COCs were visually assessed, photographed and analyzed using an OLYMPUS FV1200 FLUOVIEW scanning confocal laser microscope (OLYMPUS, Tokyo, Japan) at an excitation wavelength λ_ex_ equal to 540 nm and an emission wavelength λ_em_ equal to 580 nm. Sections from each COC were evaluated under both 20× and 40× objective lenses and scored by an observer blinded to the treatment groups. For each COC section, all cross-sectional CCs profiles (*n* = 100) were counted, and the number of TUNEL-positive cells was ascertained.

### 4.6. Determination of cAMP Concentration in EAC-Treated COCs Subjected to 3D-IVM

The concentration of cAMP was measured using Cyclic AMP Direct ELISA (DRG MEDTek, Warsaw, Poland) in compliance with the manufacturer’s protocol. Following termination of 3D-IVM and simultaneous exposure to selected endocrine disruptors, FAB-encapsulated COCs derived from all groups were washed twice with CaCl_2_- and MgCl_2_-deprived PBS and placed in alginate-dissolving buffer comprising 0.055 M sodium citrate and 0.15 M NaCl for 30 min at 37 °C. Subsequently, COCs were centrifuged at 1100 rpm at 4 °C for 10 min. Supernatant was removed, and COCs were rinsed twice with PBS and centrifuged again. For protein isolation, all collected COCs (~46 COCs per experimental group) were lysed with the provided Sample Diluent. After 10 min incubation at room temperature, the suspension was sonicated. Samples were centrifuged at 1100 rpm at 4 °C for 10 min, and the supernatant was assayed directly. First Standard (150 pM/mL) was prepared by the addition of 30 μL of cAMP stock solution to 270 μL of Sample Diluent. Standards 2–6 (50, 16.67, 5.56, 1.85 and 0.617 pM/mL) were prepared by serial dilutions. The regular format of the assay protocol was used to quantitatively estimate the intracytoplasmic cAMP concentration. Prior to carrying out an assay, the Plate Primer was added to all wells that were used. Nonspecific binding (NSB) wells and Zero standard were filled with Sample Diluent (75 μL and 50 μL). All samples were run in duplicate and, before initiating an assay, were diluted four times. Afterwards, cAMP Conjugate and cAMP Antibody were added to all wells (except for NBS wells). To mix all reagents, the sides of the plate were gently tapped, and the plate was shaken at room temperature for 2 h. In the next step, all reagents were aspirated, and each well was washed four times with ELISA Wash Buffer. Then, the plate was incubated at room temperature for 30 min with 3,3′,5,5′-Tetramethylbenzidine (TMB) Substrate. To stop the reaction, to each well, Stop Solution was added. All analyses were performed in duplicate using Labtech LT-4500 Microplate Reader (Labtech International Ltd., Heathfield, East Sussex, UK) at a detection wavelength λ equal to 450 nm. Free ELISA software (elisaanalysis.com) was used to calculate cAMP concentration using built-in 4-Parameter Logistic Regression. All obtained results were multiplied by the dilution factor.

### 4.7. Quantitatively Ascertaining the Intracytoplasmic Glutathione Concentration in 3D-IVM-Produced COCs Exposed to the Selected Endocrine Disruptors

The Glutathione Colorimetric Detection Kit (Invitrogen™, Thermo Fisher Scientific) was used to quantify the intracellular concentration of glutathione (GSH) in Vnz-, Ndn- and CsA-treated or untreated COCs. All necessary reagents were provided by the manufacturer. To obtain the cell lysate, the pools of COCs derived from all experimental groups (at a quantity of approximately 42 COCs per group) were transferred to 1.5 mL Eppendorf tubes filled with 5% 5-sulfosalicylic acid dihydrate (SSA; Sigma-Aldrich). They were subsequently sonicated thrice for 5 s at 50 V, followed by incubation for 10 min at 4 °C. Afterwards, all samples were centrifuged at 14,000 × *g* at a temperature of 4 °C for 10 min, and the supernatants were used for further analysis. All samples were diluted 4 times in Assay Buffer, followed by de novo quadruple dilution in Sample Diluent. Standards were prepared according to the manufacturer’s instructions. A total of 50 µL of the test sample or standard was consecutively added to the wells of a 96-well plate, followed by addition of 25 µL of Colorimetric Detection Reagent to each well. In the final step, 25 µL volumes of the reaction mixture, which was composed of NADPH (reduced/hydrogenated isoform of nicotinamide adenine dinucleotide phosphate) Concentrate, Glutathione Reductase Concentrate and Assay Buffer, were added. The plate was incubated for 20 min at room temperature. Measurement of intracytoplasmic GSH concentration was achieved at a detection wavelength λ equal to 405 nm using a Labtech LT-4500 Microplate Reader (Labtech International Ltd.).

### 4.8. Transmission Electron Microscope Analysis of Porcine COCs Undergoing 3D-IVM and Simultaneous EDC Treatment

Ultrastructural alterations in COCs were detected by transmission electron microscopy (TEM) as described previously [168]. Briefly, control, Vnz-, Ndn-, and CsA-treated COC samples were fixed with 2.5% glutaraldehyde (Polysciences Inc., Warrington, PA, USA), 0.067 M cacodylate buffer (pH 7.3) and 3 mM calcium chloride for 2 h at 4 °C. Samples were then washed in cacodylate buffer with calcium chloride at 4 °C for 2 h and post-fixed in 2% osmium tetroxide (Sigma-Aldrich) with 0.8% potassium ferrocyanide (Chempur^®^, Piekary Śląskie, Poland) in the same buffer for 1 h at 4 °C. After dehydration in a series of ethanol and finally in propylene oxide, the material was embedded in PolyBed 812 epoxy resin (Polysciences Inc., Warrington, PA, USA). Ultrathin (80 nm thick) sections were contrasted with uranyl acetate and lead citrate according to standard protocols and analyzed with a Jeol JEM 2100 transmission electron microscope (TEM) (JEOL, Tokyo, Japan) at a maximum voltage output of 80 kV.

### 4.9. The Live Cell-Based Assay of Mitochondrial Metabolic Activity Assisted by the Seahorse XFp Analyzer

The COC bioenergetics were determined using the Seahorse XFp Analyzer (Agilent, Boston, MA, USA) kindly provided by Perlan Technologies (Poland). All assays were programmed (designed) in XF data acquisition Wave 2.6.1 software (Agilent, Boston, MA, USA). In each experiment, 3 baseline measurements were taken prior to the addition of any compound/substrate/inhibitor, and at least 3 response measurements were taken after the addition of each compound. The oxygen consumption rate (OCR) and extracellular acidification rate (ECAR) are reported as absolute rates (pM/min for OCR and mpH/min for ECAR). While sensor cartridges were hydrated (overnight) and calibrated (XF Calibrant), cell plates were incubated at 37 °C for 30 min prior to starting an assay. All experiments were performed at 37 °C in non-CO_2_ conditions. Detailed protocols and their justification can be found at https://www.agilent.com/en/product/cell-analysis/how-torun-an-assay (accessed from 1 June 2020 to 1 May 2021). Additionally, detailed protocols were previously published [35,169,170].

#### Seahorse XF Measurement of ECAR and OCR Using Seahorse XF Cell Mito Stress Test

Following 3D-IVM under the conditions of Vnz, Ndn or CsA exposure, COCs were suspended in sterile (i.e., 0.2 µm syringe strainer-filtered) sodium bicarbonate-depleted Hank’s Balanced Salt Solution (NaHCO_3_-free HBSS; Gibco, Waltham, MA, USA). The NaHCO_3_-free HBSS medium was enriched with 1 mM sodium pyruvate (Sigma-Aldrich), 2 mM L-Glutamine (Sigma-Aldrich), 10 mM D-glucose (Lonza Bioscience, Basel, Switzerland) and 5 mM HEPES (Sigma-Aldrich) and adjusted to pH 7.4 with 0.1 N NaOH (Sigma-Aldrich). The buffer factor of assay media was validated prior to experiments and was equal to 2.9 mM/pH. In the next step, COCs were plated (6 COCs/well) in 180 µL on Agilent Seahorse 8-well XFp Cell Culture Miniplate and allowed to settle/adhere for 30 min at 37 °C. The performance of Seahorse XF Cell Mito Stress Test resulted in real-time and noninvasive measurements of ECAR and OCR that were positively correlated with the levels of acidification stemming, to the largest extent, from such intramitochondrial metabolic activities as biochemical reactions of glycolysis and ATP biosynthesis, respectively. Measurements were continued for 1 h and consisted of (i) a sample mixing time (each 1 min long) and (ii) a data acquisition period of 57 min. The latter consisted of 3 cycles, with a waiting time before each measurement lasting for 15 min.

### 4.10. Assessment of Mitochondrial Distribution Pattern in 3D-IVM-Generated COCs Exposed to the Selected EDCs

MitoTracker™ Orange CM-H2TMRos (MtOR-CM; Invitrogen™, Thermo Fisher Scientific) was used to visualize mitochondria in COCs according to the method developed by Romek et al. [171]. Briefly, a 0.5 mM MtOR-CM working solution was prepared on the basis of TCM 199 medium. The MM was removed from the culture plate of COCs and replaced with 0.5 mM MtOR-CM solution. Following 30 min incubation at a temperature of 38 °C and in an atmosphere of 5% CO_2_ in humidified air, COCs were washed twice in Ca^2+^- and Mg^2+^-deprived PBS and subsequently fixed in 4% PFA for 5 min at room temperature. Afterwards, the COCs were de novo rinsed twice with PBS and counterstained with DAPI diluted in VECTASHIELD Antifade Mounting Medium (Vector Laboratories). The images were visualized under a 60× oil objective on an OLYMPUS FV1200 FLUOVIEW confocal laser microscope with the use of an excitation wavelength of λ_ex_ = 554 nm and an emission wavelength of λ_em_ = 576 nm. The quantification of the cell fluorescence intensity was conducted with the NIH ImageJ software.

### 4.11. Detection of Mitophagy Incidence in COCs Subjected to 3D-IVM and Simultaneously Treated with the Selected EACs

A Mitophagy Detection Kit^®^ (Dojindo, Kumamoto, Japan), which was composed of Mtphagy Dye^®^ and Lyso Dye^®^, was used to confirm the occurrence of mitophagy-dependent processes that were induced by exposing in vitro maturing COCs to the selected endocrine disruptors. The procedure was conducted according to the previously reported protocol [172] with an additional modification. Briefly, after 24 h preculture of porcine COCs, 100 nM Mtphagy Dye was added to the maturation medium. Following 30 min incubation (in the dark), COCs were washed twice with TCM 199 medium. Subsequently, the medium intended for 3D-IVM culture of COCs was supplemented with either Vnz, Ndn or CsA. After 3D-IVM culture for a further 48 h was terminated, the COCs were washed with TCM 199 medium, followed by treatment of ex vivo–matured COCs with the addition of 1 M Lyso Dye to each well. In the next step, the multi-well plate filled with meiotically matured COCs was incubated for an additional 30 min followed by washing with HBSS (Thermo Fisher Scientific). The oocytes and CCs that stemmed from COCs undergoing IVM under 3D culture conditions were imaged using an OLYMPUS FV1200 FLUOVIEW scanning confocal laser microscope at an emission wavelength λ_em_ equal to 650 nm, as prescribed by the manufacturer. The fluorescence intensity was quantified using the NIH ImageJ software (National Institutes of Health, Bethesda, MD, USA).

### 4.12. Statistical Analysis

Statistical analysis was performed using Statistica v.13.1 software (Stat-Soft, Inc., Tulsa, OK, USA). The experiments aimed at the 3D-IVM culture of porcine COCs exposed or not exposed to the selected EDCs were carried out in quintuplicate (*n* = 5). Brown–Forsythe test for homogeneity of variance, Bartlett’s test for normality and one-way ANOVA followed by Dunnett’s post hoc test were used to estimate intergroup variability not only among experimental EDC treatments but also between experimental EDC treatments and their control counterparts. All data are presented as the overall mean ± standard error of the mean (SEM), and intergroup differences were considered to be statistically significant at the 95% confidence level (* *p* < 0.05).

## 5. Conclusions

The objective of our current investigation was to verify an overarching research hypothesis. The results highlighted the negative impact of the selected endocrine-active compounds, which encompassed a pesticide (Vnz), an anabolic steroid (Ndn) and an immunosuppressant (CsA), on not only the developmental competences of oocytes and viability of CCs but also the molecular quality and mitochondrial metabolic activity of porcine COCs. The obtained results provide strong scientific evidence that both Vnz and Ndn decrease the developmental competence of oocytes and activate the processes of apoptosis in CCs. Furthermore, it is noteworthy that Vnz accelerates the maturation process in immature oocytes through both increased ROS production and the augmented transcriptional activity of the *CCNB1* gene encoding cyclin B1. It also triggers the onset and progression of proapoptotic events in CCs by activating the transcription factor FOXO3, which regulates the mitochondrial apoptosis pathway. In turn, Ndn inhibits the maturation of oocytes by prompting the molecular scenarios responsible for: (1) lessening the ATP synthase-mediated generation of ATP within the mitochondria; (2) enhancing the accumulation of lipid droplets and subsequently (3) intensifying the adenylate cyclase-mediated biosynthesis of cAMP from ATP and finally (4) increasing the intraooplasmic cAMP concentration. However, due to the simultaneous increase in the expression of TNF-β and HSP27 proteins in CCs, Ndn may induce the processes of their neoplastic transformation, which brings about the disruption of intercellular communication within the whole COC. Finally, our research robustly supports that CsA does not interfere with the nuclear and cytoplasmic maturation of the oocytes. Nonetheless, the oocytes exposed to CsA are not supplied with the appropriate abundance of ATP, which would ensure the proper development of the embryo. This finding arises from a powerful scientific assessment confirmed by the high incidence of mitophagy in CCs, the direct consequence of which is insufficient ATP biogenesis within the intramitochondrial oxysomes in porcine COCs undergoing CsA treatment under 3D-IVM conditions.

Cumulatively, extensive efforts were undertaken in our present study to thoroughly decipher and quantitatively estimate a wide spectrum of molecular determinants affecting Vnz/Ndn/CsA-evoked impairments of mitochondrial activity in porcine 3D-IVM-derived oocytes intended to be used as not only meiotically matured ova able to be fertilized by standard IVF or ICSI but also nuclear recipient cells for SCNT. These efforts might allow for devising reliable and feasible approaches applied to either precisely identify or felicitously predict biomarkers correlated with the complete synchronization of the processes of meiotic, epigenomic and cytoplasmic maturation in oocytes originating from COCs grown extracorporeally in the innovative 3D culture model developed in the present research. Development and optimization of the 3D-IVM model mimicking in vivo maturation conditions that are characterized by a totally coordinated scenario of nuclear, epigenomic and ooplasmic maturation of nuclear recipient oocytes might contribute to a considerable increase in the efficiency of somatic cell cloning in pigs and other mammalian species. Furthermore, the ex vivo models designed based on 3D-IVM of porcine oocytes exposed to Vnz, Ndn or CsA might create empirical foundations to devise a panel of novel preclinical and clinical therapies aimed at ovary-specific regenerative medicine and ovarian tissue engineering strategies. The latter might be dedicated to sub-fertile or infertile female patients, whose oocytes display severe acquired insufficiency in the processes of meiotic maturation. This insufficiency can arise not only from the separate or combined intensive exposure of human ovarian follicles to Vnz, Ndn and CsA but also from the strong cytotoxic properties of these potent EACs/EDCs.

## Figures and Tables

**Table 1:** Primers used for RT-qPCR.

Gene	F/R	Primer Sequence (5′→3′)	T_m_ (°C)	Reference
*GAPDH*	F	CCCACGAGCACACCTCAGAA	55.9	[161]
	R	TGCAGCCTGTACTCCCGCT	55.4	[161]
*GPX4*	F	ATTCTCAGCCAAGGACATCG	51.8	[162]
	R	CCTCATTGAGAGGCCACATT	51.8	[162]
*FOXO3*	F	GGGGAGTTTGGTCAATCAGA	51.8	[163]
	R	TGCATAGACTGGCTGACAGG	53.8	[163]
*SIRT3*	F	CAGCGGCATTCCAGACTTCA	53.8	[164]
	R	GTCCCAACCATCAAACTTTCCA	53.0	[164]
*CASP3*	F	GAGGCAGACTTCTTGTATGC	51.8	[162]
	R	CATGGACACAATACATGGAA	47.7	[162]
*LC3*	F	CCGAACCTTCGAACAGAGAG	53.8	[162]
	R	AGGCTTGGTTAGCATTGAGC	51.8	[162]
*CDC2*	F	TGGGCACTCCCAATAATGAA	49.7	[165]
	R	TCCAAGCCATTTTCATCCAA	47.7	[165]
*CCNB1*	F	GCTCCAGTGCTCTGCTTCTC	55.9	[165]
	R	ACAAACTTTATTAAAAGTAAATAAGTG	47.6	[165]
*ATP5A1*	F	AGTTGCTGAAGCAAGGACAGTAT	53.5	[161]
	R	GTGTTGGCTGATAACGTGAGAC	54.8	[161]

## Data Availability

Not applicable.

## Abbreviations

ΔΨm	Mitochondrial membrane/transmembrane potential
3D	Three-dimensional
3D-IVM	Three-dimensional in vitro maturation
AAS	Anabolic androgenic steroids
ARTs	Assisted reproductive technologies
ATP	Adenosine-5′-triphosphate
ATP5A1	ATP synthase F1 subunit α
bad	Bcl2-associated agonist of cell death
Bcl-2	B-cell lymphoma 2
BIM	Bcl2-interacting mediator of cell death
cAMP	Cyclic adenosine 3′,5′-monophosphate
CCNB1	Cyclin B1 gene
CCs	Cumulus cells
CDC2	Cell division cycle 2
Cdc25	Cell division cycle 25
CDK1	Cyclin-dependent kinase 1
cg	Cortical granule
COCs	Cumulus–oocyte complexes
CsA	Cyclosporin A
DSCN	Donor somatic cell nuclei
DAPI	4′,6-Diamidino-2-phenylindole
dUTP	2′-Deoxyuridine-5′-triphosphate
EA	Early autophagosome
EACs ECAR	Endocrine-active compounds Extracellular acidification rate
EDCs	Endocrine-disrupting chemicals
FAB	Fibrin-alginate hydrogel bead
FasL	Fas ligand
FasR	Fas receptor
FBS	Fetal bovine serum
FITC	Fluorescein-5-isothiocyanate
FOXO3a	Forkhead box O3a
FSH	Follicle-stimulating hormone
GAPDH	Glyceraldehyde 3-phosphate dehydrogenase
GSH GV	Glutathione Germinal vesicle
GVBD	Germinal vesicle breakdown
hCG HepG2	Human chorionic gonadotropin Human hepatocarcinoma-derived cell lines
HSP27	Heat shock protein 27
ICSI	Intracytoplasmic sperm injection
IVF	In vitro fertilization
IVM	In vitro maturation
IVP	In vitro embryo production
LA	Late autophagosome
LC3	Microtubule-associated protein 1A/1B light chain 3β
LD	Lipid droplets
LH	Luteinizing hormone
MII	Metaphase II
MM	Maturation medium
MPF	Maturation/meiosis-promoting factor
mPTP	Mitochondrial permeability transition pore
MQ	Molecular quality
mRNA	Messenger RNA
mt	Mitochondria
MtOR	MitoTracker Orange
NADPH	Nicotinamide adenine dinucleotide phosphate (reduced form)
Ndn	Nandrolone
NSCLC	Non-small cell lung cancer
OCR PB	Oxygen consumption rate Polar body
pFF	Porcine follicular fluid
PMSG	Pregnant mare serum gonadotropin
RA	Relative abundance
qRT-PCR	Quantitative reverse transcriptase real-time polymerase chain reaction
ROS	Reactive oxygen species
RT	Reverse transcription
SCNT	Somatic cell nuclear transfer
SIRT3	Sirtuin 3
TNF-β	Tumor necrosis factor-β
TUNEL	Terminal deoxynucleotidyl transferase-mediated dUTP nick-end labelling
Vnz	Vinclozolin
ZP	Zona pellucida
